# Genomic characterization of the most barotolerant *Listeria monocytogenes* RO15 strain compared to reference strains used to evaluate food high pressure processing

**DOI:** 10.1186/s12864-020-06819-0

**Published:** 2020-07-02

**Authors:** Ilhan Cem Duru, Margarita Andreevskaya, Pia Laine, Tone Mari Rode, Anne Ylinen, Trond Løvdal, Nadav Bar, Peter Crauwels, Christian U. Riedel, Florentina Ionela Bucur, Anca Ioana Nicolau, Petri Auvinen

**Affiliations:** 1 Institute of Biotechnology, University of Helsinki, Helsinki, Finland; 2 Department of Process Technology, Nofima – Norwegian Institute of Food, Fisheries and Aquaculture Research, N-4068 Stavanger, Norway; 3 Department of Chemical Engineering, Norwegian University of Science and Technology (NTNU), Trondheim, Norway; 4 Institute of Microbiology and Biotechnology, Ulm, University, Albert-Einstein-Allee 11, 89081 Ulm, Germany; 5 Faculty of Food Science and Engineering, Dunarea de Jos University of Galati, Galati, Romania

**Keywords:** High-pressure processing (HPP), Foodborne pathogen, CRISPR, PacBio, Comparative genomics, Pan-genome

## Abstract

**Background:**

High pressure processing (HPP; i.e. 100–600 MPa pressure depending on product) is a non-thermal preservation technique adopted by the food industry to decrease significantly foodborne pathogens, including *Listeria monocytogenes*, from food*.* However, susceptibility towards pressure differs among diverse strains of *L. monocytogenes* and it is unclear if this is due to their intrinsic characteristics related to genomic content. Here, we tested the barotolerance of 10 different *L. monocytogenes* strains, from food and food processing environments and widely used reference strains including clinical isolate, to pressure treatments with 400 and 600 MPa. Genome sequencing and genome comparison of the tested *L. monocytogenes* strains were performed to investigate the relation between genomic profile and pressure tolerance.

**Results:**

None of the tested strains were tolerant to 600 MPa. A reduction of more than 5 log_10_ was observed for all strains after 1 min 600 MPa pressure treatment. *L. monocytogenes* strain RO15 showed no significant reduction in viable cell counts after 400 MPa for 1 min and was therefore defined as barotolerant. Genome analysis of so far unsequenced *L. monocytogenes* strain RO15, 2HF33, MB5, AB199, AB120, C7, and RO4 allowed us to compare the gene content of all strains tested. This revealed that the three most pressure tolerant strains had more than one CRISPR system with self-targeting spacers. Furthermore, several anti-CRISPR genes were detected in these strains. Pan-genome analysis showed that 10 prophage genes were significantly associated with the three most barotolerant strains.

**Conclusions:**

*L. monocytogenes* strain RO15 was the most pressure tolerant among the selected strains. Genome comparison suggests that there might be a relationship between prophages and pressure tolerance in *L. monocytogenes*.

## Background

*Listeria monocytogenes* is a well-known foodborne pathogen that may cause listeriosis, a severe infection with high hospitalization rate which can be fatal in humans and some animal species [1]. Although being a relatively rare foodborne disease, increasing numbers of listeriosis cases have been reported in the EU/EEA countries since 2008 [2]. *L. monocytogenes* is generally found in agricultural, aquacultural, and food processing environments [3]. Listeriosis outbreaks have been associated with consumption of contaminated food, such as meat, fruits, vegetables, milk, cheese, and fish [3, 4]. The food industry has to process food in such a way to meet food safety microbiological criteria (Commission Regulation (EC) No 1441/2007 [5]), which include the presence in limited numbers (100 cfu/g) or absence of *L. monocytogenes* (absence in 25 g product). Food processing aimed at *L. monocytogenes* inactivation is challenging due to its pronounced ability to adapt to different environments, and to survive under various stress conditions [6]. In addition, it is important to preserve the food quality and nutritional value during food processing. High pressure processing (HPP) is an alternative to thermal processes to reduce the concentration of pathogens, including *L. monocytogenes*, and maintain a high quality of the product. Only very small or no quality changes, such as loss of color, nutritional value, flavor, and texture, have been reported for HPP products [7] . Several studies showed that HPP products, such as meat, milk, juice, vegetables, and sauce are well accepted by the consumer, and reported that HPP preserves the taste [8]. Depending on the food products, pressures currently used in the food industry are between 100 and 600 MPa, with a holding time ranging from one to several minutes [9]. HPP has been tested and reported as an effective processing method for several products including fruits (and fruit juices) [10], meat [11], cheese [12], fish [13] and vegetables [14].

It has been shown that HPP has varying effects on different target organisms. For example, a pressure of 300 MPa is sufficient to inactivate most Gram-negative bacteria, while more than 400 MPa is needed for an inactivation of Gram-positive bacteria [15, 16]. It is also known that pressure tolerance can differ even among strains of the same species. Differences in pressure tolerance have been reported for strains of several species including *Cronobacter sakazakii* (previously *Enterobacter sakazakii*) [17], 24 different serotypes of *Salmonella enterica* [18], and *L. monocytogenes* [19, 20]. In *L. monocytogenes* strains, pressure tolerance varies between 300 and 500 MPa [19]. To our knowledge, genomic profiling and comparison has not been performed for barotolerant and barosensitive *L. monocytogenes* strains. In this study, we selected 10 *L. monocytogenes* strains, isolated from either foods, food processing environments or clinical sources, to test their tolerance towards HPP at 400 and 600 MPa, compare their genomes, and investigate whether genetic traits may be associated with pressure tolerance.

## Results

### Pressure treatment and reduction of viable cell counts

**Fig. 1:**
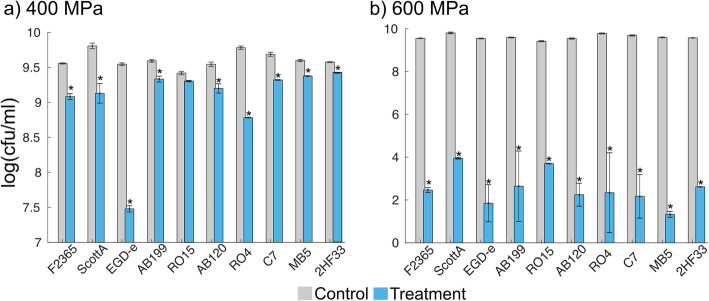
Viable cell counts bar chart. Viable cell counts log_10_ (cfu/ml) of untreated controls (gray bar) and samples treated (blue bar) for 1 min at 400 MPa (**a**) or 600 MPa (**b**). Data are presented as mean of 6 replicates. Error bars represent standard deviation (ANOVA; *, *p* < 0.02)

Reduction of viable cell counts (colony forming units, cfu) after pressure treatment at 400 and 600 MPa for 1 min showed that pressure tolerance differs between strains. The variance in log_10_ colony forming units per milliliter (cfu/ml) of the treated samples was significantly larger (p < 0.02, see ANOVA results in Fig. 1a) compared to control, indicating a high variance in the level of tolerance of *L. monocytogenes*. At 400 MPa for 1 min, the 10 strains exhibited an average log_10_ reduction of 0.57 cfu/ml, ranging from 0.05 log_10_ cfu/ml for the most barotolerant strain (RO15) to 2.07 log_10_ cfu/ml for the most pressure sensitive strain (EGD-e). Similar results were obtained at 600 MPa. Here, the 10 strains exhibited an average log_10_ cell number reduction of 7.06 cfu/ml, with a range of 5.42 to 8.27 log_10_ cfu/ml (Table 1). Strain RO15 was also the most barotolerant strain based on an initial screening including several other *L. monocytogenes* strains (Supplementary text 1, Table S1, Figure S1).

**Table 1:** Reduction in viable cell counts after 1 min 400 and 600 MPa pressure treatment. Table shows the average reduction in log_10_ (cfu/ml) of selected *L. monocytogenes* strains after 1 min at 400 or 600 MPa pressure compared to untreated control samples (n = 6 to 9 treated/untreated samples per strain). Standard deviation is shown in brackets

	Log_10_ reduction compared to control -Δlog_10_ (cfu/ml) (standard deviation)	
Strain	400 MPa	600 MPa
RO15	0.05 (0.14)	5.42 (0.15)
2HF33	0.15 (0.08)	6.97 (0.10)
MB5	0.22 (0.14)	8.27 (0.27)
AB199	0.26 (0.12)	6.95 (1.16)
AB120	0.35 (0.16)	7.30 (0.59)
C7	0.37 (0.17)	7.52 (0.88)
F2365	0.47 (0.16)	7.10 (0.26)
ScottA	0.68 (0.57)	5.86 (0.39)
RO4	1.00 (0.13)	7.70 (1.24)
EGD-e	2.07 (0.33)	7.49 (0.70)

One-way analysis of variance (ANOVA), comparing the pressure (400 MPa) treated samples of each strain to the control samples, showed that the mean log_10_ cfu/ml for treated samples was significantly lower than the control for all strains (p < 0.02), except for strain RO15 (p = 0.15) (Fig. 1a). The same statistical analysis for HPP at 600 MPa indicated that all strains including strain RO15 had significantly (p < 0.01) lower log_10_ cfu/ml in treated samples compared to controls (Fig. 1b).

### Genome sequencing, general features and RNA-Seq

**Fig. 2:**
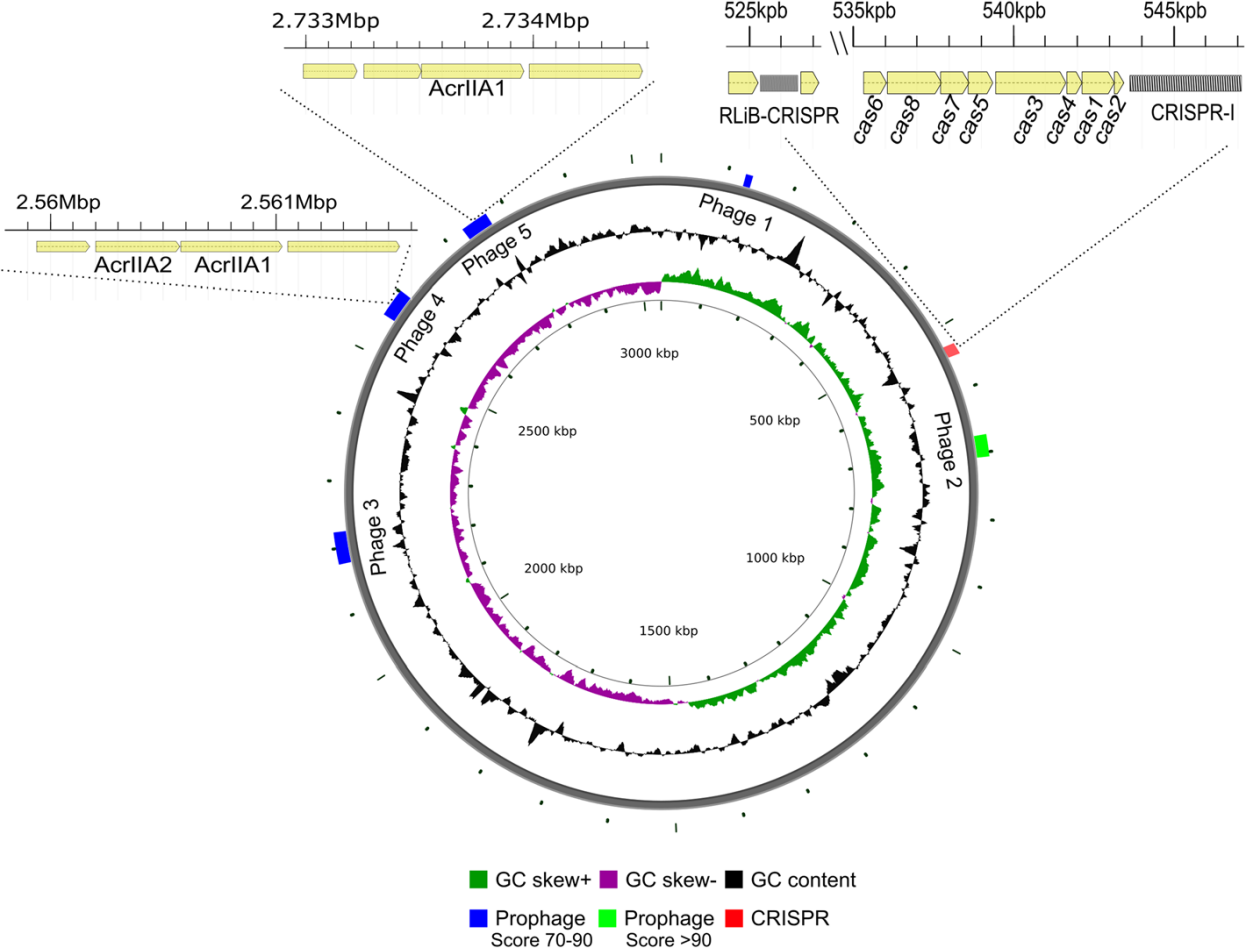
Circular map of the *L. monocytogenes* strain RO15 chromosome with predicted prophage regions, CRISPR region and anti-CRISPR genes. Five prophage regions were predicted in the chromosome of *L. monocytogenes* strain RO15 using the PHASTER tool, as shown in figure with blue and green color. Two anti-CRISPR gene regions were annotated within the prophage region 4 and 5. The RliB-CRISPR/Cas system was located at the position 525–527 kbp, following CRISPR-I system located at the position 535–547 kbp with *cas*1–8 genes and a CRISPR array with 54 spacers

We sequenced the genomes of seven *L. monocytogenes* strains for which no genome sequences were available in public databases. Genomes of strains RO15, 2HF33, MB5, AB199, AB120, C7, and RO4 were sequenced using Illumina MiSeq equipment and strain RO15 was sequenced using Pacbio RSII. Sequences of these seven strains were assembled and three additional strains (ScottA, F2365 and EGD-e, genome sequences of which are available in public databases) were used for comparative genome analysis. Assembly of PacBio long reads of strain RO15 resulted in one continuous 3,042,507 bp sized chromosome (Fig. 2) at an average 308-fold sequencing coverage. In addition to the chromosome, a contig with a complete circular prophage sequence of 38,811 bp was obtained, which was also found as part of the chromosome (2729417–2,770,759 bp). All genomes had a similar size, GC content and number of coding sequences (CDS) (Table 2).

**Table 2:** General genome summary of selected *L. monocytogenes* strains. The table shows the genome size, genome sequence based prediction of clonal complex (CC), multilocus sequence typing (MLST) based on 7 loci, core genome MLST (cgMLST), lineage, and serotypes of selected strains

Strain	Size, Mbp (CDS)	CC	Serotype	MLST	cgMLST	Lineage	Genome Source	Source	Reference
RO15	3.04 (3022)	CC155	1/2a	155	11632	II	This study (Complete)	Herring with spices	[21]
2HF33	3.11 (3098)	CC121	1/2a	121	3328	II	This study (Contigs)	Salmon filleting section	[22]
MB5	2.97 (2958)	CC7	1/2a	7	9444	II	This study (Contigs)	Salmon gutting machine	[﻿22﻿]
AB199	2.99 (2907)	CC204	1/2a	204	6821	II	This study (Contigs)	Drain/processing room	[21]
AB120	3.00 (2924)	CC204	1/2a	204	6821	II	This study (Contigs)	Sausage filler machine	[21]
C7	3.09 (3051)	CC8	1/2a	8	999	II	This study (Contigs)	Salmon gutting machine	[22]
F2365	2.91 (2808)	CC1	4b	1	2	I	GenBank: AE017262.2	Mexican-style cheese	[23]
ScottA	3.02 (2966)	CC2	4b	290	94	I	GenBank: CM001159.1	Clinical isolate	[24]
RO4	2.88 (2806)	CC20	1/2a	20	13510	II	This study (Contigs)	Dry cured salami	[21]
EGD-e	2.94 (2875)	CC9	1/2a	35	1	II	GenBank: AL591824.1	Rabbit tissue	[25, 26]

The serotype of the strains was reported in previous studies [21–25] (Table 2). Although RO15 was reported as a serovar 4b strain using multiplex PCR in a previous study [21], our genome-based prediction suggests that it belongs to PCR-serogroup 1/2a. Sequences for *ORF2110* and *ORF2819* primers used for identification of serovar 4b strains [25] were not found in the genome of strain RO15 based on our sequence match analysis, whereas sequences for primers targeting *lmo0737*, which are indicators of serovar 1/2a strains [25] were present. Multilocus sequence typing (MLST) based on 7 loci, core genome MLST (cgMLST; based on Moura scheme [27]), and clonal complex (CC) of the strains were also assigned based on the genome sequences (Table 2). Lineage assignment showed that only ScottA and F2365 belonged to lineage I and all other strains were members of lineage II (Table 2).

In addition to genome sequencing, transcriptome analysis using RNA-seq for strains RO15 and ScottA provided a basic view of transcriptional activity of the genomes. Differential expression analysis indicated that virulence genes and heat shock genes were upregulated after high pressure treatment in strain ScottA (Table S2).

### Methylated DNA motifs

High PacBio sequencing coverage allowed us to analyze DNA methylation modification motifs in strain RO15 in addition to assembling the genome. Using the long read modification data, 11 methylated sequence motifs were detected in the genome (Table 3). None of the detected motifs had a partner motif, i.e. a reverse-complementary sequence, but all detected motifs were only partially modified in the genome with less than 50% methylated motifs. While most of the motifs have already been deposited in the REBASE database [28] DADGYATYA, WNNTVVGCNTWNH, AHNBAACA, AGNNARNWW were novel, i.e. they have not been described as potential methylation sites previously. None of the detected motifs have been reported as recognition sequence motif for a restriction enzyme in the REBASE database.

**Table 3:** A summary of motifs. The table shows the detected methylated motifs and the total number of motifs in the RO15 genome

Motifs	Type	% Motifs Detected	# of Motifs Detected	# of Motifs in Genome	mean Coverage
ADGYACYTV	m6A	44.03%	420	954	149.9
ADDTGGCA	m6A	30.97%	455	1469	148.5
TVVARARG	unknown	22.20%	1835	8265	148.8
ANNYASYA	m6A	22.10%	3289	14,879	149.0
DADGYATYA	m6A	21.02%	326	1551	147.7
WNNTVVGCNTWNH	unknown	18.14%	582	3208	149.2
AHNBAACA	m6A	13.36%	839	6282	150.2
AGNNARNWW	m6A	9.85%	2225	22,596	148.6
TNNNDNNH	unknown	9.22%	114,413	1,240,313	148.9
TNNNCRVHNH	unknown	7.59%	6652	87,598	149.0
TVNNNNNG	unknown	3.26%	7834	240,556	149.6

We predicted one type II cytosine-5 DNA methyltransferase gene (OCPFDLNE_00657), and three type II N4-cytosine or N6-adenine DNA methyltransferase genes (OCPFDLNE_02168, OCPFDLNE_02626, OCPFDLNE_02808) in the strain RO15 genome. In addition, genes for a type IV methyl-directed restriction enzyme (OCPFDLNE_00324) and type II restriction enzymes (OCPFDLNE_00658, OCPFDLNE_02625, OCPFDLNE_02807) were predicted. These predicted methyltransferases and restriction enzymes had significant (e-value <1E-50) BLASTP hits in the REBASE protein sequences database [28]. However, their recognition sequences are unknown. OCPFDLNE_02625 and OCPFDLNE_02807 type II restriction enzyme genes in prophage regions were expressed based on RNA-seq data.

### Genomic comparison of the selected strains

In the genome assembly we also identified a circular phage as an independent contig in strain RO15. While looking after phage-originating genome parts, prophage prediction revealed five prophage regions (10.7 kb to 47.9 kb sized) in the RO15 genome (Fig. 2). Prophage (region) 5 had the same sequence as the separate circular phage. RNA-Seq count data suggested that most of the genes of prophage 1,3 and 5 in RO15 were transcribed. Subsequently, the prophage prediction was performed for all strains, which showed that all strains had at least one or more prophage regions in the genome with a maximum of six prophage regions in strain C7 (Table 4). One of the predicted prophage regions in ScottA was also transcribed based on the RNA-seq data.

**Table 4:** Comparison of CRISPR/Cas systems, anti-CRISPR genes, and prophage regions amongst the selected *L. monocytogenes* strains

Strain	Average reduction of log_10_ (cfu/ml) at 400 MPa	CRISPR/Cas systems	Number of spacers	Self-targeting spacer predicted	anti-CRISPR gene predicted	Number of prophage predicted
RO15	0.05	RliB-CRISPR, CRISPR I	64	yes	AcrIIA1, A2	5
2HF33	0.15	RliB-CRISPR, CRISPR I, CRISPR II	62	yes	AcrIIA1, A2, A3, A4	5
MB5	0.22	RliB-CRISPR, CRISPR II	39	yes	AcrIIA1, A2, A3	5
AB199	0.26	RliB-CRISPR	3	no	no	1
AB120	0.35	RliB-CRISPR	3	no	no	1
C7	0.37	RliB-CRISPR	7	no	no	6
F2365	0.47	RliB-CRISPR	3	no	no	1
ScottA	0.68	RliB-CRISPR	3	no	no	3
RO4	1.00	RliB-CRISPR	5	no	AcrIIA1, A2, A3	1
EGD-e	2.07	RLIB-CRISPR	4	no	AcrIIA1, A2, A3	2

**Fig. 3:**
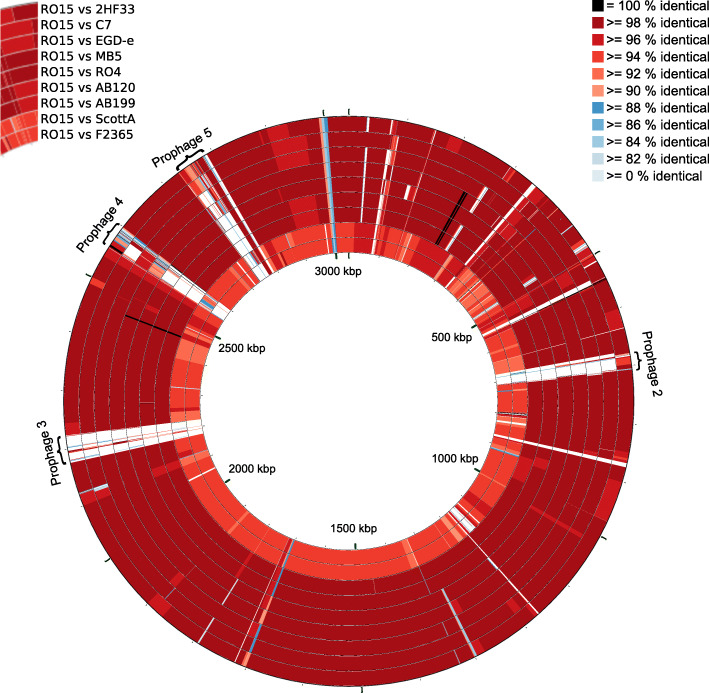
Genome comparison of the selected nine strains mapped against strain RO15. The genomes were aligned using BLAST and visualized as ring figures. Each ring represents the genome alignment to strain RO15. From inside to outside, strains are located according to increasing hits of high percent identity to strain RO15 (Order from inside to outside: strain F2365, ScottA, AB199, AB120, RO4, MB5, EGD-e, C7, and 2HF33. Color represents BLAST hit identity percentage). Red colored BLAST hits represent 90–98% identity, blue colored BLAST hits represent 80–90% identity. Black colored BLAST hits represent 100% identity. White gaps represent < 80% identity

Genome alignment of the selected nine strains against the strain RO15 (Fig. 3) showed large sequence gaps between strain RO15 and the other strains. The gaps were mainly related to prophage regions with prophage region 3 being specific for strain RO15. Prophage region 2 was only seen in the strain 2HF33. Prophage region 4 was partially seen in strains 2HF33, EGD-e, MB5 and ScottA. Similarly, prophage region 5 was partially seen in strains 2HF33, C7, and RO4. In addition, strains ScottA and F2365 had less aligned regions compared to the other strains. Average nucleotide identity (ANIb) results showed that ANIb scores of strains ScottA and F2365 with other strains were lower than 95 (Table S3). To study how the large sequence gaps are localized in the other publicly available genomic sequences of *L. monocytogenes*, all 200 complete *L. monocytogenes* genomes in RefSeq database [29] were downloaded and aligned to strain RO15 (Figure S2). The visualization of the alignment showed that prophages 2, 3 and 4 were seen only in the minority of the *L. monocytogenes* strains, while prophages 1 and 5 were seen in the majority of the *L. monocytogenes* strains. The comparison indicated that these prophage regions were common locations of variance between *L. monocytogenes* strains.

**Fig. 4:**
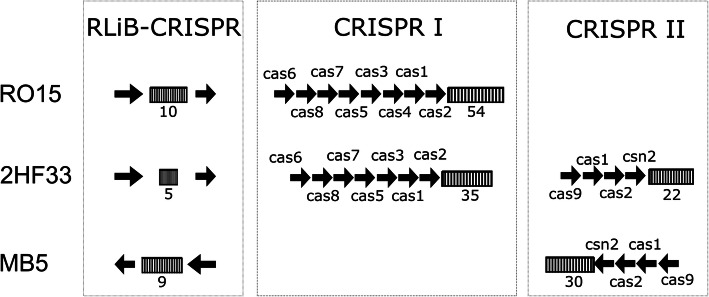
Visualization of CRISPR systems in strains RO15, 2HF33, and MB5. The figure shows a representation of CRISPR systems in strains RO15, 2HF33, and MB5. These three strains contained more than one CRISPR system, while the rest of the selected strains in the study contained only the RliB-CRISPR system. Arrows represent genes, rectangles represent repeat/spacer arrays, the numbers below the rectangles indicate the number of spacers in the array. For simplification, sizes of the arrows do not correspond to the actual size of the genes

Annotation of CRISPR systems revealed that the RliB-CRISPR system was seen in all the strains, which is in line with previous RliB-CRISPR system studies [30, 31]. Interestingly, CRISPR I or CRISPR II system genes were present only in strains RO15, 2HF33, and MB5 (Fig. 4), which exhibited the lowest reduction in log_10_ cfu/ml with the 400 MPa pressure treatment (Table 1). Number of spacers in these strains were also significantly (p < 0.001) higher compared to barosensitive strains. The alignment of the spacer sequences of the CRISPR systems back to the genome itself revealed that only strains RO15, 2HF33, and MB5 contained self-targeting spacers with 100% identity. As expected, spacer sequences were aligned to the prophage regions, except one spacer sequence in RO15, which aligned to the *addB* gene (OCPFDLNE_02427) located in the chromosome encoding an ATP-dependent helicase.

Using prediction tools we detected anti-CRISPR genes in the prophage regions (Table 4) of strains RO15, 2HF33, MB5, RO4, and EGD-e. Homologues of all four previously annotated *Listeria* anti-CRISPR genes, i.e. *acrIIA1*, *acrIIA2*, *acrIIA3*, and *acrIIA4* [32], were seen in strain 2HF33. Gene *acrIIA4* was not seen in strains MB5, RO4, and EGD-e, but the rest of the anti-CRISPR genes were present. RO15 contains two copies of *acrIIA1* (OCPFDLNE_02770, OCPFDLNE_02583) and *acrIIA2* (OCPFDLNE_02582) (Fig. 2) and we observed expression of these anti-CRISPR genes in strain RO15.

As antibiotic resistance and pressure tolerance were linked in a previous study [19], we also searched antibiotic resistance genes in all strains to identify relation of antibiotic resistance genes and pressure tolerance. The same antibiotic resistance gene families were detected in all the 10 strains (Table S4). Multiple sequence alignment of the detected antibiotic resistance genes and the following average distance tree generation showed that amino acid sequence of the *norB* gene (encoding for a quinolone resistance protein) (OCPFDLNE_03068) was slightly different in barotolerant strain RO15 compared to the other strains (Figure S3). Similarly, for the *lin* gene (encoding for a lincomycin resistance protein) (OCPFDLNE_00980) amino acid difference was seen for strains 2HF33 and RO15 (Figure S3).

**Fig. 5:**
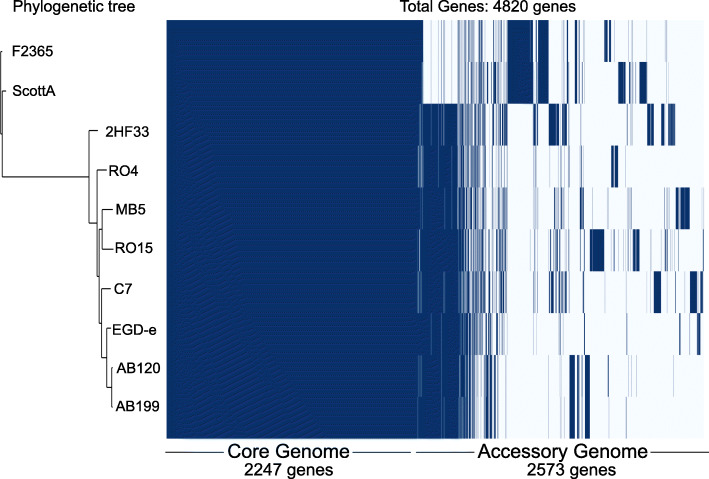
Pan-genome comparison of selected strains. A phylogenetic tree was created based on core genome alignments of the selected strains. The matrix shows presence (blue) and absence (white) of core and accessory genes. Core genes are found in all strains, accessory genes are found in at least one strain

Roary pan-genome pipeline suggested that the 10 *L. monocytogenes* strains contain a total of 4820 orthologous gene clusters. Of these genes, 2247 were core genes found in all studied strains and 2573 genes were accessory genes found in at least one strain. The core genome was used for constructing a phylogenetic tree, which indicated that the two serovar 4b strains (ScottA and F2365) are closely related to each other and cluster separately from the serovar 1/2a strains in the phylogenetic tree. In addition, there was also a clear difference in accessory genome for serovar 4b strains compared to serovar 1/2a strains (Fig. 5).

To test any significant association between pressure tolerance and (clusters of) genes in the accessory genome, we performed a pan-genome wide association analysis using Scoary v1.6.16 [33]. Based on the pairwise comparison of reduction in log_10_ (cfu/ml) against strain RO15 using Student’s t-test (Table S5), a statistically significant (p < 0.05) difference compared to RO15 was seen in all strains except MB5 and 2HF33. Therefore, strains RO15, 2HF33, MB5 were used as barotolerant strains for the pan-genome association analysis. Scoary results showed that 13 gene clusters (Table S6) had p-values < 0.01 for the association with barotolerant strains, i.e., the genes were only seen in barotolerant strains. Of these, 10 gene clusters were located in the prophage regions, but most of the genes were annotated as hypothetical proteins (Table S6). These prophage genes were also searched for in 200 complete *L. monocytogenes* strains and found in some strains (Table S6). Interestingly, genes OCPFDLNE_02579 and OCPFDLNE_02580 were only seen in strains that harbored anti-CRISPR genes, Cas type I or II CRISPR system and self-targeting spacers (Table S6).

## Discussion

HPP is commonly used in the food industry to inactivate foodborne pathogens and spoilage organisms. Depending on the type of food product, up to ~ 600 MPa pressure is applied [9]. It has also been shown that combinations of HPP and biocontrol agents, such as virulent bacteriophages and bacteriocins, have synergistic effects in the inactivation of the target pathogens in food [34, 35]. The pressure tolerance level varies between different species, strains, and even isolates [16, 19, 36, 37]. Here, we tested pressure tolerance of 10 *L. monocytogenes* strains, compared their genomes and predicted genome features related to pressure tolerance.

Pressure treatment using 600 MPa for 1 min caused more than 5 log_10_ reduction in all selected strains, which is generally considered sufficient for food safety regulations. According to the Food Safety and Inspection Service (United States Department of Agriculture), 5 log_10_ reduction is considered a full lethality treatment for *L. monocytogenes* [38]. This suggests that the strains used in this study were relatively sensitive to 600 MPa pressure treatment. Similarly, an earlier study reported that pressure treatment at 500 MPa for 10 min provided sufficient reduction in viable counts to reach desired level of safety for all except one *L. monocytogenes* strains tested [19]. However, the lower pressure levels, such as 400 MPa, would be more relevant for industrial applications, therefore, we focused more on the results obtained with the 400 MPa treatment. Among the strains tested, RO15 was the most barotolerant strain when processed at both 400 and 600 MPa, which is in line with the initial selection process of strains (Supplementary text 1). Based on ANOVA, RO15 was the only strain for which no statistically significant reduction was observed with the 400 MPa treatment (Fig. 1a). Therefore, we defined the strain RO15 as barotolerant. It has been shown that the level of inactivation by HPP can be affected by the chemical composition of the food product. Some food products, such as milk and cheese, have baroprotective effects on microorganisms during HPP treatment [39, 40]. However, experiments in this study were performed using TSBYE broth and thus do not take into account the effects of food composition on HPP. Temperature during pressure treatment can have an effect on microbial inactivation depending on treatment time, and it has been shown that increasing the temperature from 25 °C to 35 °C has a significant effect on the viability loss of *L. monocytogenes* [41]. In this study, temperature during pressure treatment increased up to 33 °C for 400 MPa treatment and 38 °C for 600 MPa treatment due to adiabatic heating. However, since treatment time was only 1 min, the effect of temperature rise was expected to be minimal.

Serotypes of the selected strains were shown in the earlier studies based on agglutination method, multiplex-PCR and genome sequencing [21, 23–25] (Table 2). Here, we predicted the serotype of all strains based on their genome sequences. This confirmed the previous serotyping of all strains except RO15, which was reported to be a serovar 4b strain based on multiplex-PCR [21]. Serotype 4b strains should contain serovar 4 marker genes (*ORF2110* and *ORF2819*) and lack *lmo0737* marker gene sequence as described by Doumith et al. [25]. However, according to the genome sequence, serovar 4 marker genes were absent, and a *lmo0737* homologue was present suggesting that the serotype of strain RO15 is indeed PCR-serogroup 1/2a. The phylogenetic tree that we created based on the core genome showed that there is considerable genetic variation between serovars. Serovar 4b strains (ScottA and F2365) clustered separately from the other strains (Fig. 4). Strain RO15 was not clustered together with other serovar 4b strains hence these results supported our gene-based prediction of PCR-serogroup 1/2a of strain RO15.

PacBio sequencing provides not only the genome sequence but also methylation data [42], which gives an opportunity to analyze restriction-modification systems and their recognition motifs. In this study, we did not observe a genuine methylated motif based on PacBio sequencing data of strain RO15. Similarly, PacBio methylation data of nine other published *L. monocytogenes* strains without genuine methylated motifs were also seen in REBASE PacBio list database [28], which showed that it is not uncommon to have only partially methylated motifs in *L. monocytogenes*.

Previous studies based on *L. monocytogenes* strain ScottA and LO28 barotolerant isolates showed that there is a phenotypic and genotypic variation between barotolerant isolates [36, 37, 43], which indicates that there are variety of factors which can lead to pressure tolerance. Therefore, it is a challenge to link a genomic profile with pressure tolerance in different strains. Nevertheless, pan-genome association analysis results suggest that there was a genotypic difference between barotolerant and barosensitive strains. A total of 13 genes were significantly (p < 0.01) associated with barotolerant strains. Interestingly, most of the barotolerant strain associated genes (Table S6) were located in the prophage regions in the studied genomes. In addition, extension search of these genes within all complete *L. monocytogenes* strain genomes showed that some phage genes (OCPFDLNE_02579 and OCPFDLNE_02580) were only seen in strains that contain inactivated CRISPR/Cas system (Table S6). It has been shown that prophages can provide increased biofilm formation and can be beneficial for stress coping in both *L. monocytogenes* and *Escherichia coli* [44, 45]. We also observed that barotolerant strains harbored a slightly higher number of prophages compared to barosensitive strains (based on average number of prophages in barotolerant and barosensitive strains). Therefore, we believe that prophages might play a role in barotolerance in *L. monocytogenes*. However, further phage gene deletion or reintroduction studies are needed to verify relation between phages and barotolerance.

It was also interesting to see that two or more CRISPR-Cas systems, self-targeting spacers, and anti-CRISPR genes were seen together only in the three most barotolerant strains (RO15, MB5, and 2HF33). Since the existence of a self-targeting spacer indicates that the activity of the CRISPR/Cas systems is inhibited [46], it can be predicted that anti-CRISPR proteins detected in barotolerant strains inactivate CRISPR/Cas system. Previous studies have also shown that anti-CRISPR proteins are able to inhibit several types of CRISPR systems [32, 47]. The observed active transcription of anti-CRISPR genes in RO15 prophages based on RNA-seq may also support this prediction. It has been shown that bacteria tend to lose CRISPR/Cas systems, since they can be harmful to the host itself. Immunopathological effects through partially matching spacers were reported unless the phage carries anti-CRISPR genes [48]. The anti-CRISPR genes found in prophages and inhibition of the CRISPR/Cas system may be the reason why barotolerant strains carry type I or type II CRISPR/Cas systems. We observed that more than 50% of the *L. monocytogenes* strains that harboring type I or type II CRISPR/Cas system also had anti-CRISPR genes (Table S6). CRISPR/Cas systems can have alternative roles in bacteria, such as roles in pathogenesis or role in modulating biofilm formation [49], however the role of inhibited CRISPR/Cas system is not known. More studies are required to support the relation between inhibited CRISPR/Cas systems and barotolerance.

RNA-seq revealed the general transcriptome profile of strains RO15 and ScottA. Significantly upregulated heat-shock genes after pressure treatment (chaperone and clp protease genes) in ScottA (Table S2) might indicate that proper folding of proteins plays an important role in cell recovery after pressure treatment. A previous study also showed that the expression levels of Clp proteins were significantly higher in mutant HPP-tolerant ScottA isolate compared to wild type ScottA [43].

A previous study concluded that antibiotic resistant *L. monocytogenes* strains are more tolerant to pressure at 400 MPa [19]. Here, annotation and gene comparison results did not show any strain-specific antibiotic resistance gene within studied strains. Nevertheless, multiple sequence alignment of predicted antibiotic resistant genes showed that there were slight differences in amino acid sequences across the strains (Figure S3). However, it is not known that these amino acid differences cause antibiotic resistance advantage, and further studies are required to link the amino acid sequence variations of antibiotic resistance genes and pressure tolerance.

## Conclusions

In this study we noticed that barotolerance is manifested at pressures lower than 600 MPa. Strain RO15 was identified as the most barotolerant strain for 400 MPa 1 min pressure treatment. Genome sequence of seven new strains and genome comparison of 10 strains revealed that the three most barotolerant strains have CRISPR-Cas genes and anti-CRISPR genes in their genomes. In addition, the average number of prophages was slightly higher in barotolerant strains compared to barosensitive strains. Furthermore, we have predicted 10 phage genes that might be related to pressure tolerance based on the pan-genome association test. Therefore, we conclude that prophages and inhibited prophage defense systems may be linked to pressure tolerance. The observation described above may argue for the use of phage cocktails as a pretreatment before HPP and will allow to reduce pressure and holding time.

## Methods

### Strains, growth conditions and pressure treatment

Strains Scott A (CIP103575) and EGD-e (CIP107776) were obtained from Centre de Ressources Biologiques de l’Institut Pasteur, Paris, France, and strain F2365 (LMG23356) from Laboratorium voor Microbiologie, UGent, Gent, Belgium. Strains RO4, RO15, AB120 and AB199 are from Dunarea de Jos University of Galati, Romania, and have been isolated in the Promise FP7 project either from food illegally introduced to Romania or from meat processing environments. All other strains were isolated from the environment as indicated in Table 2. The strains were stored on Microbank beads with cryopreservatives (Pro-Lab Diagnostics, Toronto, Canada) at − 80 °C prior to use. The bacteria were passaged twice in tryptic soy broth (TSB) supplemented with 0.6% (w/v) yeast extract (TSBYE; Oxoid, Basingstoke, Hampshire, England). Prior to pressure treatments, bacteria were grown overnight in 50 mL TSBYE at 37 °C with rotary agitation (150 rpm), resulting in cells in early stationary phase and with a target concentration of approx. 10^9^ cfu/mL. Aliquots (10 mL) of the cultures were packaged in sous-vide plastic pouches and sealed without using vacuum, and 10 mL was transferred to 15 mL falcon tubes to serve as untreated controls.

HPP was carried out using the QFP 2 L-700 (Avure Technologies Inc., Columbus, USA). The cylindrical pressure vessel had 10 × 25.4 cm dimensions, 2 L capacity and 690 MPa upper pressure limit. Temperature of the pressure medium (water) was tracked with a K-type thermocouple located on the external surface of the samples. A holding time of 1 min was used, pressures of 400 and 600 MPa, and ambient vessel water temperature (20–22 °C). Due to adiabatic heating, water temperatures in the middle of the vessel at the end of pressure treatment had risen to 31–33 °C after the 400 MPa treatment, and 36–38 °C after the 600 MPa treatment. Straight after pressure treatment, 400 MPa pressurized and untreated samples were serially ten-fold diluted in TSBYE and plated in triplicate on tryptic soy agar with 0.6% yeast extract (TSAYE; Oxoid, Basingstoke, Hampshire, England) by using a spiral plater (Eddy Jet; IUL Instruments, Barcelona Spain). Pressurized samples at 600 MPa were additionally plated manually (100 μL) without being diluted. TSAYE plates were incubated at 37 °C for 48 h prior to counting the colonies and estimating bacterial inactivation. Two consecutive trials of the methodology were performed (Exp. 1 and 2).

### DNA extraction

DNA of each strain was extracted from 5 ml of a culture grown overnight in BHI broth at 37 °C with aeration on a rotary shaker. Wizard Genomic DNA purification kit (Promega, Madison, WI, USA) was used according to the manufacturer’s instructions.

### Library preparation, genome sequencing, de novo assembly, base modification detection and motif analysis

*L. monocytogenes* strain RO15 was sequenced using PacBio RSII (Pacific Bioscience, Menlo Park, CA, USA) and Illumina Miseq (Illumina, San Diego, CA, USA). Genomic DNA PacBio library was prepared according to the manufacturer’s Template Preparation and Sequencing Guide using DNA Template Prep Kit 2.0 and DNA/Polymerase Binding Kit P6. The Nextera XT protocol was used for the enzymatic genomic DNA fragmentation and Illumina sequencing library preparation. Pacbio reads were assembled using HGAP3 protocol [50] in SMRTPortal 2.3.0. Obtained assembly of chromosomal and phage sequences were checked and circularized using Gap4 program [51] and finally the chromosomal DNA sequence was set to start from *dnaA* gene. Cutadapt v1.8.1 [52] was used with -m 200 and -q 25 options to trim Nextera adapter sequences and quality filtering. Trimmed reads were mapped against circularized chromosomal and phage sequences using bwa-mem [53]. Short indels within homopolymeric regions were corrected using pilon v1.16 [54]. Average sequencing coverages at the whole genome level were 308X in Pacbio data and 157X in Illumina data, respectively. DNA base modifications and motifs were analyzed using Modification and Motif analysis protocol implemented in SMRTPortal 2.3.0 with default parameters.

For *L. monocytogenes* strain RO4, AB199, and AB120, Nextera DNA Library (Illumina, San Diego, CA, USA) preparation and for strain 2HF33, MB5, and C7, Nextera XT DNA Library preparation was performed. Paired-end sequencing was performed using Illumina Miseq. RTA v1.18.54 and bcl2fastq v2.17.1.14 were used for base calling, demultiplexing and converting data to fastq format. The Cutadapt v1.8.1 [52] was used with -m 200 and -q 25 options to trim Nextera adapter sequences and quality filtering. Reads aligning to PhiX genome, which was used as spike-in during Illumina sequencing, were removed by using bbmap v34.56 [55]. Spades v3.13.0 [56] with default options was used for assembling the reads and creating the contigs.

### Pressure treatment for RNA-seq experiment and RNA extraction

For both strain RO15 and ScottA, 10 ml of cells were grown in TSBYE until early stationary phase (each triplicated with new cultures). Samples were packed in sterile sous-vide pouches and packaged without applying vacuum. They were put in the refrigerator 30 min before pressure treatment. After treatment at 200 and 400 MPa for 2, 8 and 60 min at 20–22 °C, samples were transferred to sterile 15 ml Falcon tubes. From each sample, 0.5 ml were transferred to a 2 ml Eppendorf tube prefilled with 1 ml RNA Protect (Qiagen, Hilden, Germany). They were vortexed, incubated for 5 min at room temperature, and frozen at − 80 °C. Cells previously stored in RNA Protect were pelleted by centrifugation for 10 min at 5000 g. RNA extraction was performed with NucleoSpin RNA kit (Macherey-Nagel, Düren, Germany) according to manufacturer’s instructions with some modifications in the cell disruption phase. The cell pellets were suspended with 700 μl RA1 buffer and 7 μl β-mercaptoethanol (Sigma-Aldrich, Saint Louis, MO, USA). Cells were then mechanically disrupted using Lysing Matrix B tubes (MP Biomedicals, Irvine, CA, USA) and FastPrep 24 tissue homogenizer (MP Biomedicals, Irvine, CA, USA) at 6 m/s for 3 × 30 s. Cells were rested on ice for 5 min between cycles. After spinning the cells briefly and transferring the supernatant to NucleoSpin filter column, manufacturer’s protocol was followed. Quantity and quality of RNA extractions were analyzed using the Agilent 2100 Bioanalyzer and RNA 6000 Nano kit (Agilent, Santa Clara, CA, USA).

### RNA-seq library preparation

Ribosomal RNAs were removed from the total RNAs (ScottA 9.4 μl; RO15 14 μl) with Ribo-Zero rRNA Removal kit for bacteria (Illumina, San Diego, CA, USA) according to manufacturer’s instructions with 1/3 (ScottA) or 1/2 (RO15) volumes of kit solutions. rRNA-depleted RNA was purified with RNeasy MinElute Cleanup Kit (Qiagen, Hilden, Germany) according to modified protocol of the Ribo-Zero kit manual and eluted in 12 μl of RNA-free water. Eight μl of rRNA-depleted RNA was used to prepare RNA sequencing libraries with SENSE Total RNA-seq Library Prep Kit for Illumina (Lexogen, Vienna, Austria) according to manufacturer’s instructions. In reverse transcription and ligation phase, incubation time was extended to 2 h. After second-strand synthesis, purification and size-selection of the libraries were performed with 13 μl of Bead Diluent and 27 μl of Purification Solution. PCR amplification program was slightly modified by increasing the denaturation times: from 30 to 60 s in the beginning, and from 10 to 30 s during the cycles; cycle number was increased to 40. Concentration of amplified libraries was measured with Qubit fluorometer and dsDNA HS assay kit (Invitrogen, Waltham, MA, USA), and size distribution visualized with Fragment Analyzer and High Sensitivity NGS Fragment Analysis kit (Advanced Analytical, Parkersburg, WV, USA). Aliquots of amplified libraries were pooled twice for both strains. After pooling, libraries were concentrated using Amicon Ultra 100 K columns (Millipore, Burlington, MA, USA). To remove fragments under 200 bp, size selection of ScottA pools was performed using BluePippin and 2% agarose gel cassette (Sage Science, Beverly, MA, USA). In size selection of RO15 library pools, bead purification with 0.9 x AMPure XP beads (Beckman Coulter, Brea, CA, USA) and PEG/NaCl precipitation on MyOne™ carboxylic acid beads (Invitrogen, Waltham, MA, USA) were additionally used. Concentration of the pooled libraries was measured with Qubit fluorometer, and libraries were sequenced with NextSeq 500 (Illumina, San Diego, CA, USA).

### RNA-seq data analysis

Quality filtering and adapter trimming for RNA-seq reads was done using Trimmomatic v0.36 [57] using these parameters “TruSeq3-SE.fa:2:30:10 SLIDINGWINDOW:3:20 MINLEN:30”. SortMeRNA [58] was used to filter rRNA reads. Reads were mapped to the genomes using Bowtie2 [59] default settings. Aligned reads were sorted using Samtools [60]. HTSeq [61] with union mode was used for read counts. Differentially expressed gene analysis was done using DESeq2 R package [62]. Genes with adjusted p-value (padj) ≤ 0.05 and |log2 Fold Change| ≥ 1 were considered as significantly differentially expressed.

### Genome annotation, prophage prediction and genome alignment

Assembled genomes were annotated using Prokka v1.13 [63] with default options. To improve functional annotation PANNZER2 annotation web server [64] was used. CRISPR repeat regions were detected using CRISPRCasFinder version 4.2.19 [65]. Anti-CRISPR genes were annotated using BLAST alignment against known anti-CRISPR genes [32]. The prophage prediction for all genomes was done using PHASTER web tool [66]. Serotype of the selected strains were predicted by checking the marker primers [25] in the genome using EMBOSS primersearch v 6.6.0 [67]. Multilocus sequence typing (MLST) based on 7 loci (MLST), clonal complex (CC), core genome MLST (cgMLST) and lineages of strains were assigned based on MLST and the cgMLST schemes developed by Moura et al. [27, 68] by uploading the sequences to BIGSdb-Lm webserver (https://bigsdb.pasteur.fr/listeria/listeria.html). Antibiotic resistance genes were detected using Resistance Gene Identifier (RGI) tool with CARD database [69]. Heuristic neighbor-joining phylogeny tree based on concatenated sequences of the MLST gene fragments was created using FastTree v2.1 [70]. Whole genome alignment and ring figure was created using CGView Comparison Tool [71]. The ANIb scores were calculated using JSpeciesWS webtool [72].

### Pan-genome and Pan-GWAS analysis

The pan-genome analysis was done using Roary Pan-genome Pipeline [73] with default settings, to create alignment of core genes using PRANK [74] the “-e” setting was used. The core genes alignment was used for phylogenetic tree construction using FastTree v2.1 [70]. Pan-genome-wide association analysis was done using Scoary v1.6.16 [33] with default settings.

## Supplementary information

**Additional File 1 : Supplementary Text 1.***L. monocytogenes* strain selection, including **Table S1, Figure S1.**

**Additional File 2 : Table S2.** Gene count data and differentially expressed gene list for both strains RO15 and ScottA based on RNA-Seq.

**Additional File 3 : Table S3**. Table shows Average Nucleotide Identity (ANI) (based on BLAST) calculation results of each strain. For strains ScottA and F2365 the ANI score with other strains was lower than 95. ANI score between ScottA and F2365 was higher than 95. For the other strains ANI scores between each other were more than 98.

**Additional File 4 : Figure S2**. Genome comparison of 200 *L. monocytogenes* strains mapped against strain RO15. The genomes were aligned using BLAST and visualized as ring figures. a) Each ring represents the whole genome alignment to strain RO15 (100 most similar genomes (based on BLAST) were displayed in (a) for simplification), b) ring figure zoomed in the prophage 1 region, c) ring figure zoomed in the prophage 2 region, d) ring figure zoomed in the prophage 3 region, e) ring figure zoomed in the prophage 4 region, f) ring figure zoomed in the prophage 5 region. The Refseq identifiers of 200 *L. monocytogenes* strains can be seen in figure. Genome features can be seen in Table S6. Red colored BLAST hits represent 90–98% identity, blue colored BLAST hits represent 80–90% identity. Black colored BLAST hits represent 100% identity. White gaps represent < 80% identity.

**Additional File 5 : Table S4 and Figure S3. Table S4** shows identified antibiotic resistance genes and percentage identity of matching region in all strains using CARD database. **Figure S3** shows average distance trees from alignments of protein sequences of identified antibiotic resistance genes.

**Additional File 6 : Table S5.** T-test against RO15 log_10_ reduction.

**Additional File 7 : Table S6.** Barotolerant strains specific genes based on pan-genome wide association analysis. Table shows genes that have p < 0.01 based on pan-genome wide association analysis for barotolerant strains. Gene IDs of strain RO15 was used for this table. Orthologs of these genes with 95% identity were also seen in strains MB5 and 2HF33. In addition, the list of other publicly available *L. monocytogenes* genomes, where these genes were seen, are also provided.

## Data Availability

All sequencing data and assembled genomes have been deposited in the European Nucleotide Archive (ENA) under accession code PRJEB35939. Strains RO4, RO15, AB120 and AB199 are from Dunarea de Jos University of Galati, Romania, and are available upon request to anca.nicolau@ugal.ro. Strains C7, MB5, and 2HF33 are from Nofima – Norwegian Institute of Food, and are available upon request to trond.lovdal@nofima.no.

## Abbreviations

ANOVA: Analysis of variance
ANI: Average nucleotide identity
CDS: Coding sequence
cfu: Colony forming units
HPP: High pressure processing
MPa: Megapascals
MLST: Multilocus sequence typing
