# Supplementary material for: Genomic characterization of the most barotolerant Listeria monocytogenes RO15 strain compared to reference strains used to evaluate food high pressure processing
Source: BMC Genomics. 2020 Jul 2;21:455. doi: 10.1186/s12864-020-06819-0 (PMC7331262; doi:10.1186/s12864-020-06819-0)
Supplement: Supplementary file 1 — Additional File 1 : Supplementary Text 1.L. monocytogenes strain selection, including Table S1, Figure S1. [file 12864_2020_6819_MOESM1_ESM.docx]

**Additional File 1. Supplementary text for *L.* *monocytogenes* selection process**

**Additional File 1-1 Supplemental Materials and Methods**

Ten strains of *Listeria monocytogenes* from UGAL Collection were reactivated to participate to barotolerance tests having in view to find a strain resistant to high pressures used in High Pressure Processing (HPP). These ten strains were chosen to represent isolates from food, isolates from meat processing environments and different serotypes (Table S1). Reactivation of the cells took place by streaking stock cultures on Brain Heart Infusion with Agar (BHIA) and incubation of the culture medium at 37°C for 48 h. Two colonies of each strain have been transferred on liquid culture medium and cultivated at 37°C for 12 h, to obtain suspensions with the density of 10^8^ cells/mL. Suspended cells were submitted to a treatment at 400 MPa for 1 min. The treatment was performed in a HPP machine (RESATO; machine characteristics: maximum working pressure 10000 bar, 4 working vessels of 100 cc each, temperature range -20 ÷ 120°C). After treatment, cells were inoculated on BHIA and incubated for 24 h at 37°C to determine the cell number reduction.

| Strain  *L.* *monocytogenes* | Isolation source | Serotype |
| --- | --- | --- |
| AB100 | Food Salami | 1/2a (3a) |
| **RO4** | Dried cured salami | 1/2a (3a) |
| **RO15** | Herring with spices | 1/2a (3a) |
| ABS43 | Beef meat | 1/2c (3c) |
| AB80 | Sheep meat  Meat processing environments | 1/2a (3a) |
| AB24 | Floor in a cold store | 1/2a (3a) |
| **AB120** | Stainless steel table  associated to a salami/ sausage filler machine | 1/2a (3a) |
| ABS45 | Floor drain | 1/2c (3c) |
| **AB199** | Floor drain | 1/2a (3a) |
| AB204 | Tap handle of a wash hand basin | 1/2a (3a) |

**Table S1**. ***L. monocytogenes* strains from UGAL Collection submitted to barotolerance test.** The table shows isolation source and serotype of strains. Strains that written bold are selected for following study.

**Additional File 1-2 Supplemental Results**

Four of the strains submitted to the above mentioned treatment (RO15, RO4, AB120 and AB199) have been selected as being more resistant to high pressures than the other strains (log reduction ≤ 1 log) (Figure S1) and sent to Nofima to be compared with strains of *L. monocytogenes* from Nofima’s collection and with reference strains.


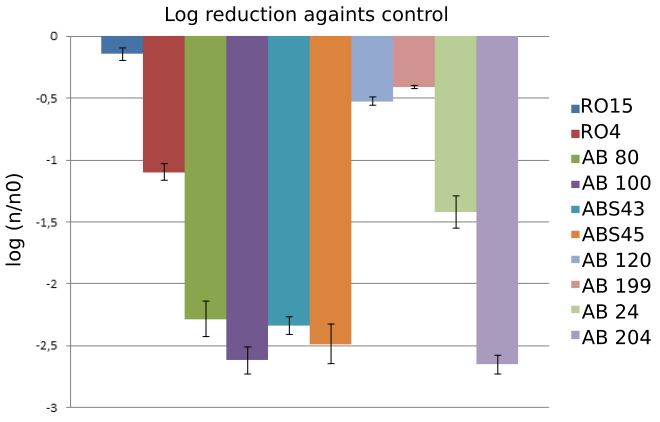


**Figure S1. Reduction of *L. monocytogenes* CFUs following a high pressure treatment (400 MPa/1 min).** Values in the graph represent the medium value of two independent assays
