# Supplementary material for: Genomic characterization of the most barotolerant Listeria monocytogenes RO15 strain compared to reference strains used to evaluate food high pressure processing
Source: BMC Genomics. 2020 Jul 2;21:455. doi: 10.1186/s12864-020-06819-0 (PMC7331262; doi:10.1186/s12864-020-06819-0)
Supplement: Supplementary file 5 — Additional File 5 : Table S4 and Figure S3. Table S4 shows identified antibiotic resistance genes and percentage identity of matching region in all strains using CARD database. Figure S3 shows average distance trees from alignments of protein sequences of identified antibiotic resistance genes. [file 12864_2020_6819_MOESM5_ESM.pdf]

			% Identity of Matching Region									
**ARO Term**	**AMR Gene Family**	**Resistance Mechanism**	**RO15**	**C7**	**ScottA**	**2HF33**	**MB5**	**F3265**	**EGD-e**	**RO4**	**AB199**	**AB120**
FosX	fosfomycin thiol transferase	antibiotic inactivation	93.98	93.98	100	92.48	92.48	100	92.48	92.48	93.98	93.98
lin	lincosamide nucleotidyltransferase (LNU)	antibiotic inactivation	98.28	98.85	97.9	98.09	98.28	97.9	100	98.09	100	100
Listeria monocytogenes mprF	defensin resistant mprF	antibiotic target alteration	100	100	99.29	100	99.88	99.42	100	100	100	100
norB	major facilitator superfamily (MFS) antibiotic efflux pump	antibiotic efflux	98.07	99.57	99.36	100	100	99.14	100	100	100	100

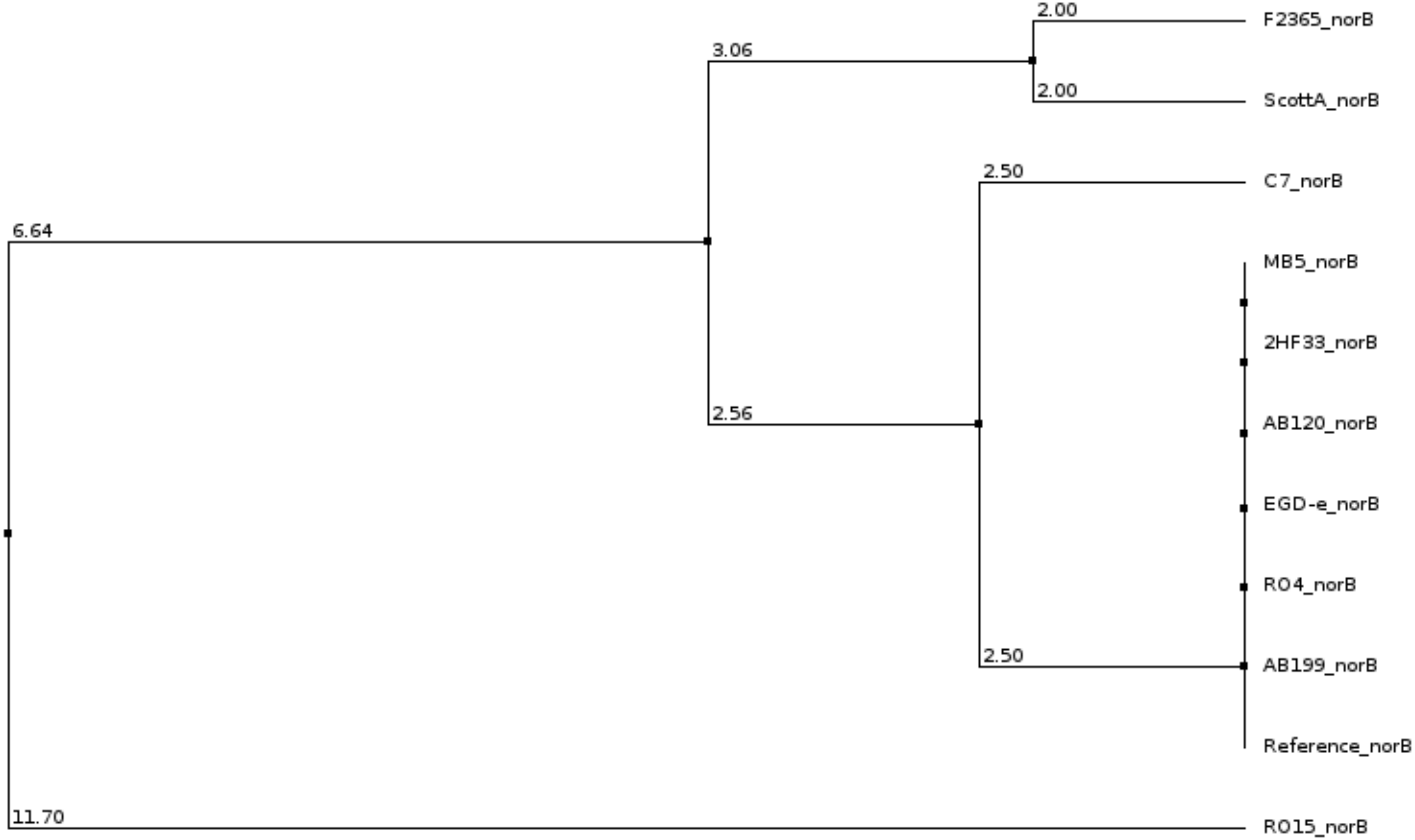
2.00
3.06
2.00
2.50
6.64
2.56
2.50
11.70
F2365_norB
ScottA_norB
C7_norB
MB5_norB
2HF33_norB
AB120_norB
EGD-e_norB
RO4_norB
AB199_norB
Reference_norB
RO15_norB

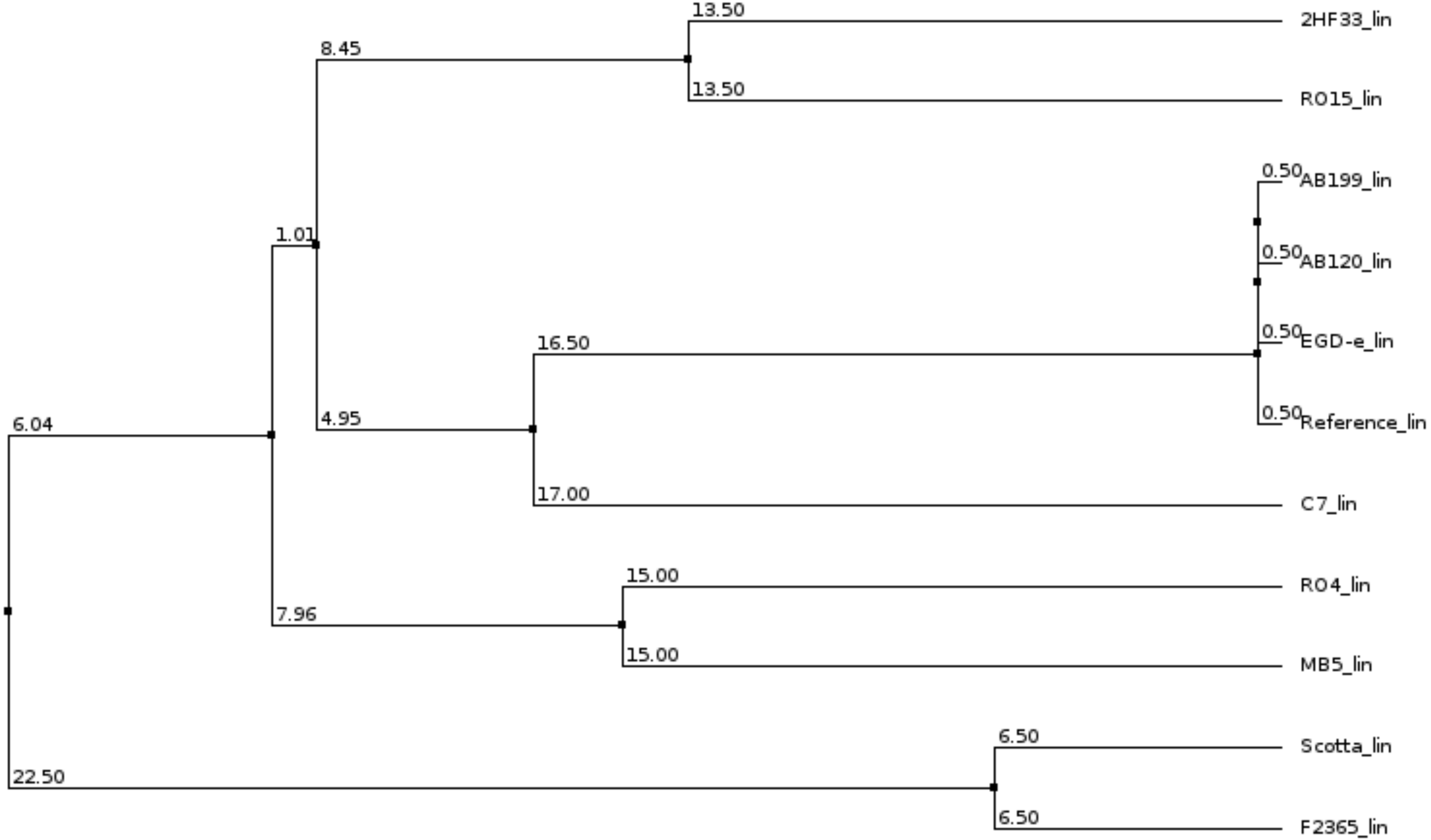

13.50
2HF33_lin
8.45
13.50
RO15_lin
0.50
AB199_lin
1.01
0.50
AB120_lin
16.50
0.50
EGD-e_lin
4.95
0.50
Reference_lin
6.04
17.00
C7_lin
15.00
RO4_lin
7.96
15.00
MB5_lin
6.50
Scotta_lin
22.50
6.50
F2365_lin

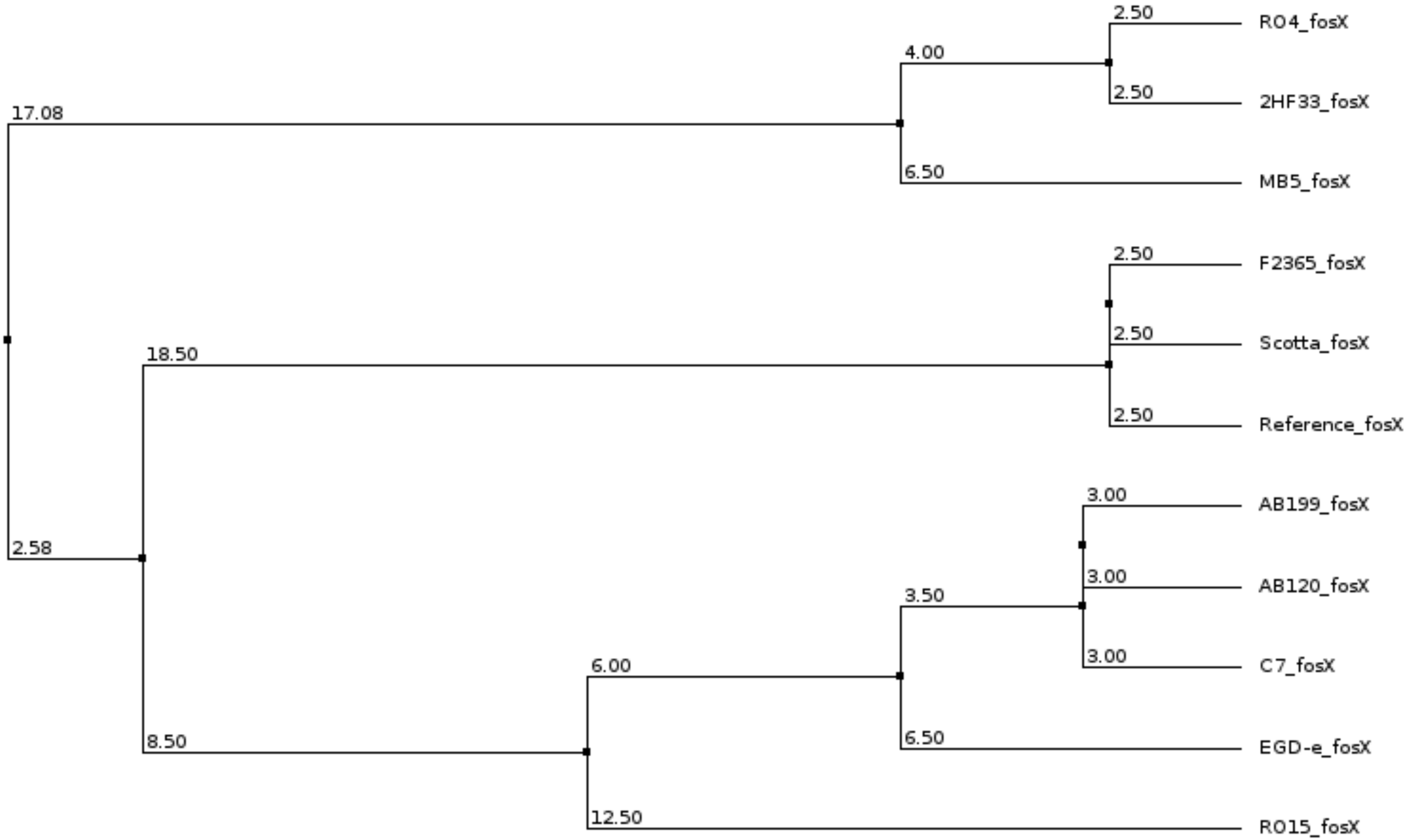
17.08
4.00
2.50
RO4_fosX
2.50
2HF33_fosX
6.50
MB5_fosX
2.58
18.50
2.50
F2365_fosX
2.50
Scotta_fosX
2.50
Reference_fosX
8.50
6.00
3.50
3.00
AB199_fosX
3.00
AB120_fosX
3.00
C7_fosX
6.50
EGD-e_fosX
12.50
RO15_fosX

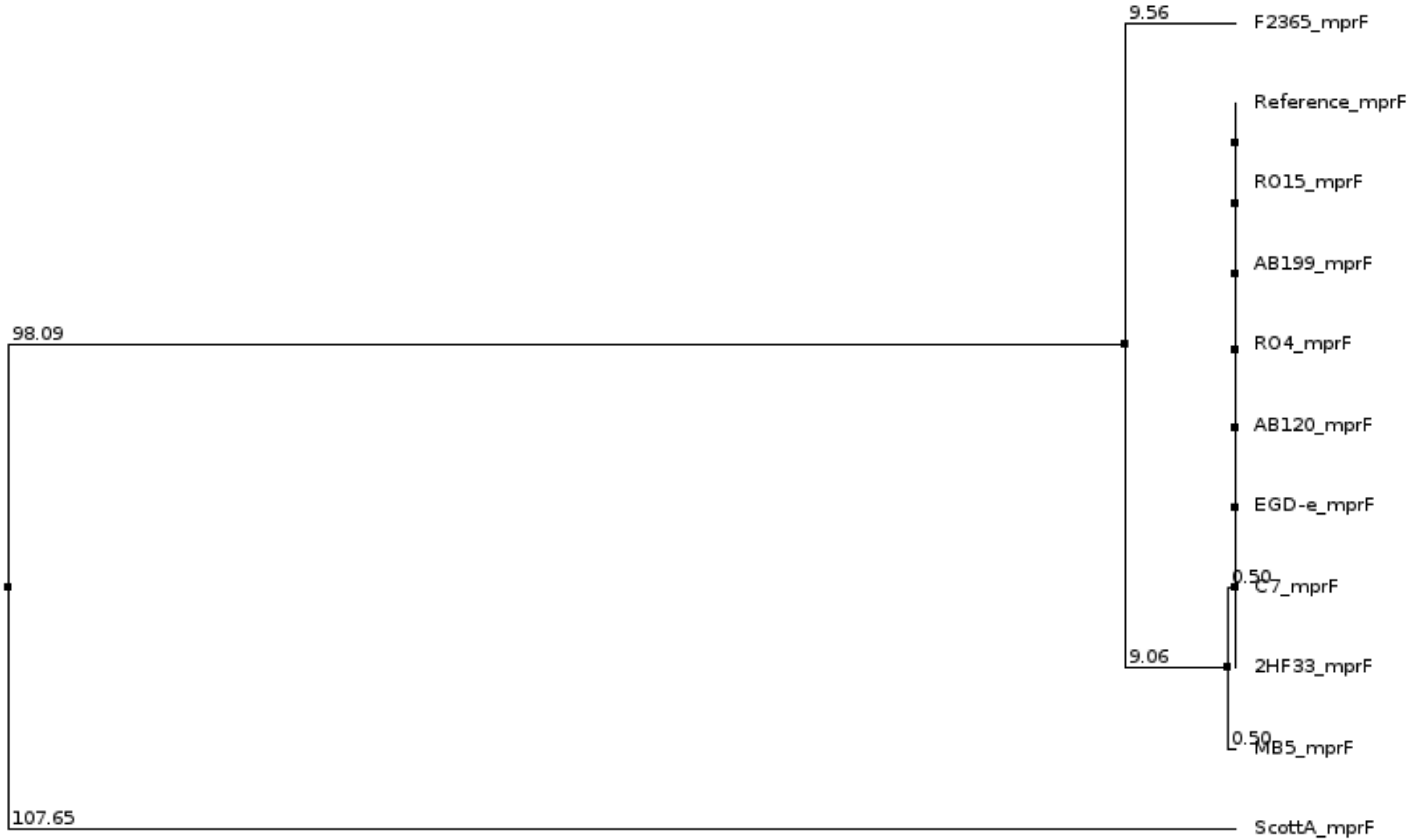
9.56
F2365_mprF
Reference_mprF
RO15_mprF
AB199_mprF
98.09
RO4_mprF
AB120_mprF
EGD-e_mprF
0.50
C7_mprF
9.06
2HF33_mprF
0.50
MB5_mprF
107.65
ScottA_mprF
