# Supplementary material for: Genomic characterization of the most barotolerant Listeria monocytogenes RO15 strain compared to reference strains used to evaluate food high pressure processing
Source: BMC Genomics. 2020 Jul 2;21:455. doi: 10.1186/s12864-020-06819-0 (PMC7331262; doi:10.1186/s12864-020-06819-0)
Supplement: Supplementary file 4 — Additional File 4 : Figure S2. Genome comparison of 200 L. monocytogenes strains mapped against strain RO15. The genomes were aligned using BLAST and visualized as ring figures. a) Each ring represents the whole genome alignment to strain RO15 (100 most similar genomes (based on BLAST) were displayed in (a) for simplification), b) ring figure zoomed in the prophage 1 region, c) ring figure zoomed in the prophage 2 region, d) ring figure zoomed in the prophage 3 region, e) ring figure zoomed in the prophage 4 region, f) ring figure zoomed in the prophage 5 region. The Refseq identifiers of 200 L. monocytogenes strains can be seen in figure. Genome features can be seen in Table S6. Red colored BLAST hits represent 90–98% identity, blue colored BLAST hits represent 80–90% identity. Black colored BLAST hits represent 100% identity. White gaps represent < 80% identity. [file 12864_2020_6819_MOESM4_ESM.pdf]

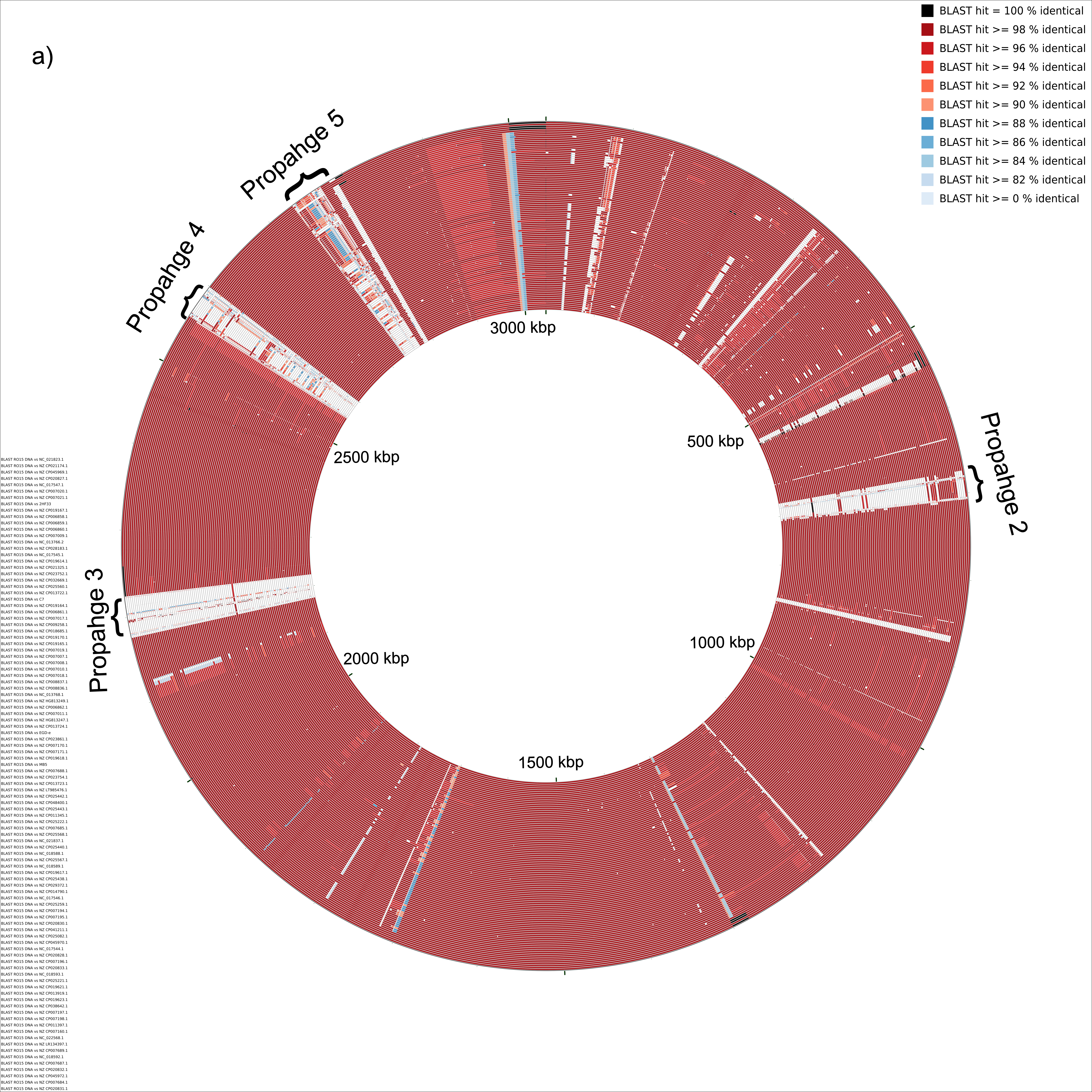
a)
BLAST hit = 100 % identical
BLAST hit >= 98 % identical
BLAST hit >= 96 % identical
BLAST hit >= 94 % identical
BLAST hit >= 92 % identical
BLAST hit >= 90 % identical
BLAST hit >= 88 % identical
BLAST hit >= 86 % identical
BLAST hit >= 84 % identical
BLAST hit >= 82 % identical
BLAST hit >= 0 % identical
Propahge 5
Propahge 4
Propahge 3
Propahge 2
3000 kbp
2500 kbp
2000 kbp
1500 kbp
1000 kbp
500 kbp

b) Prophage 1: 125824-136550 bp

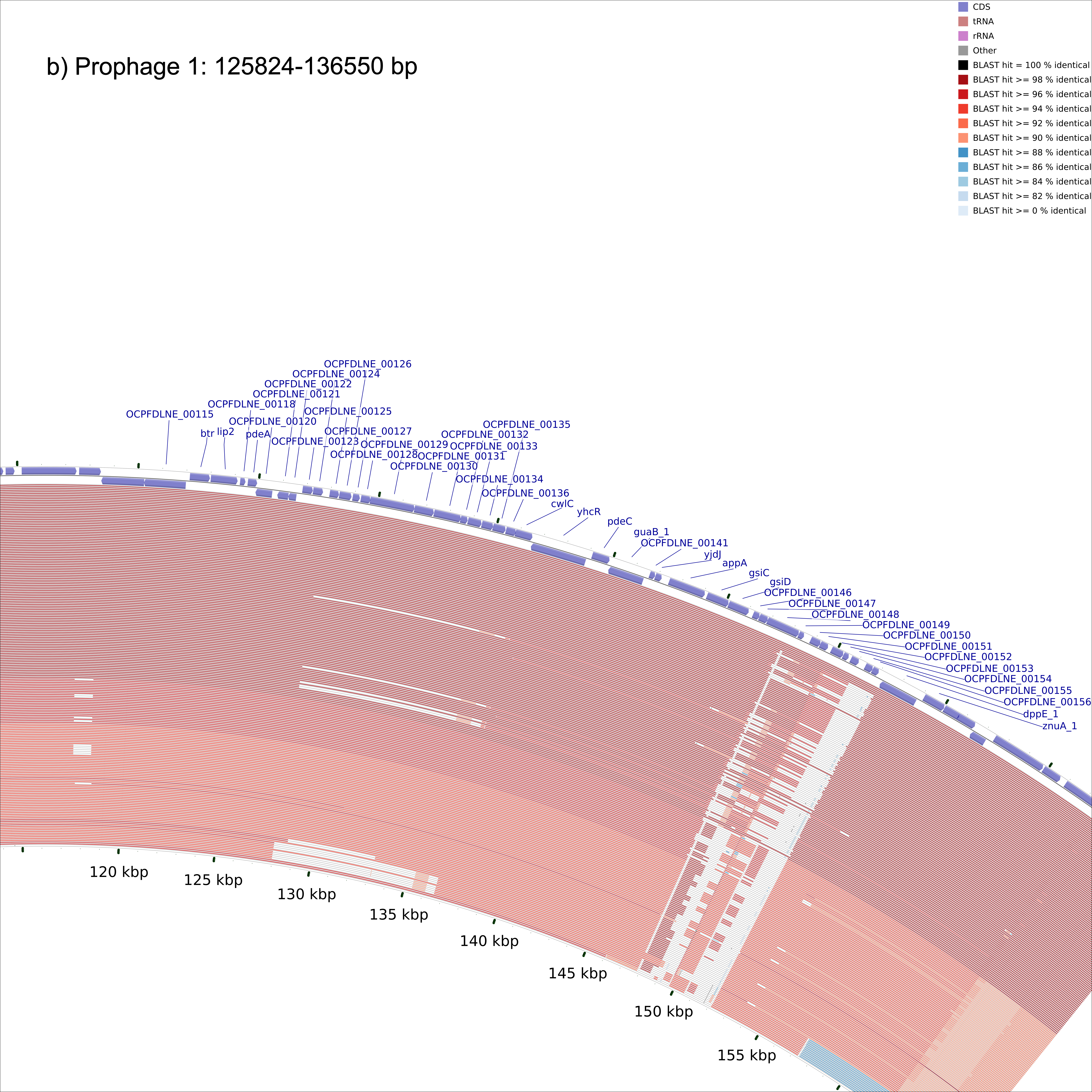

c) Prophage 2: 673631-706883 bp

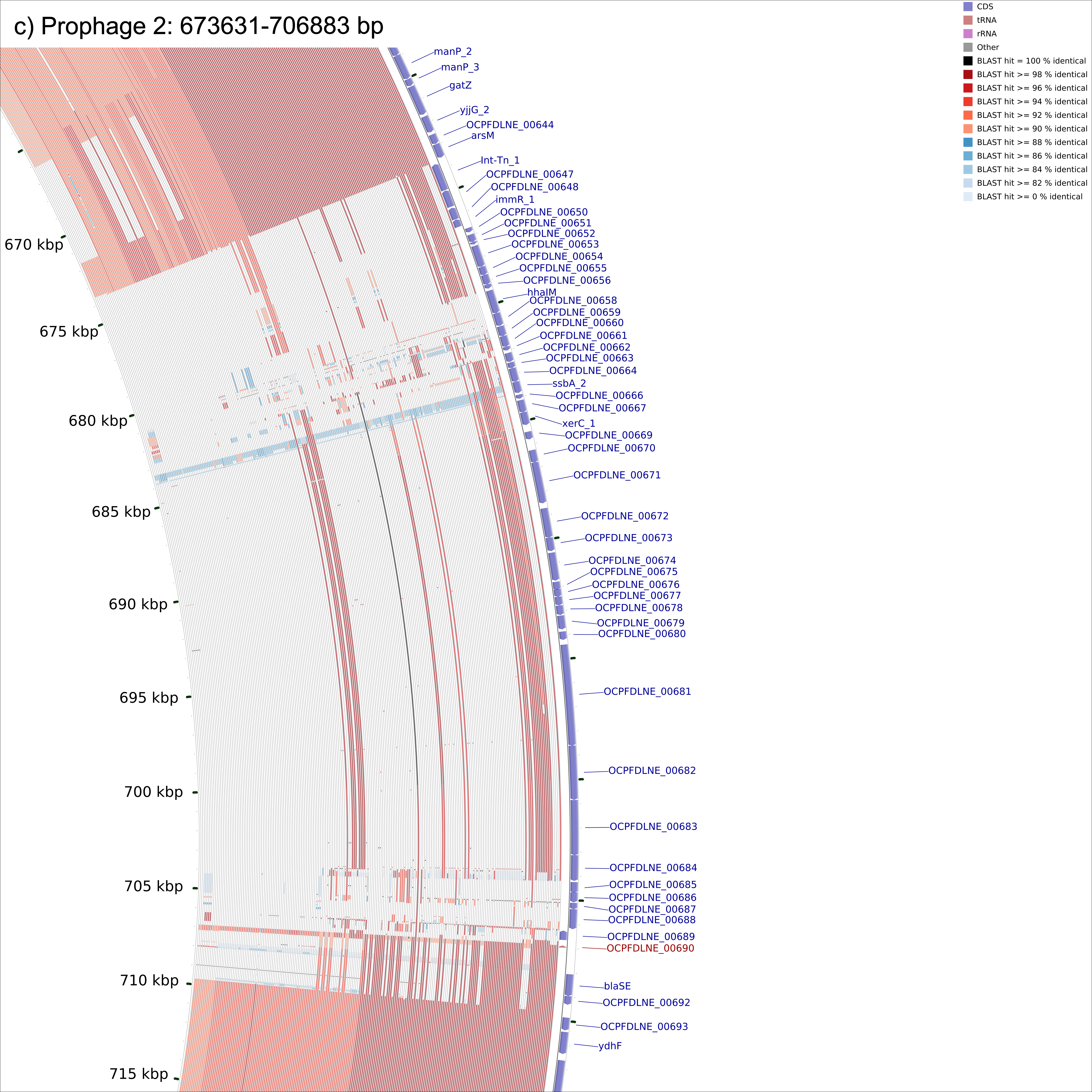

d) Prophage 3: 2175980-2223958 bp

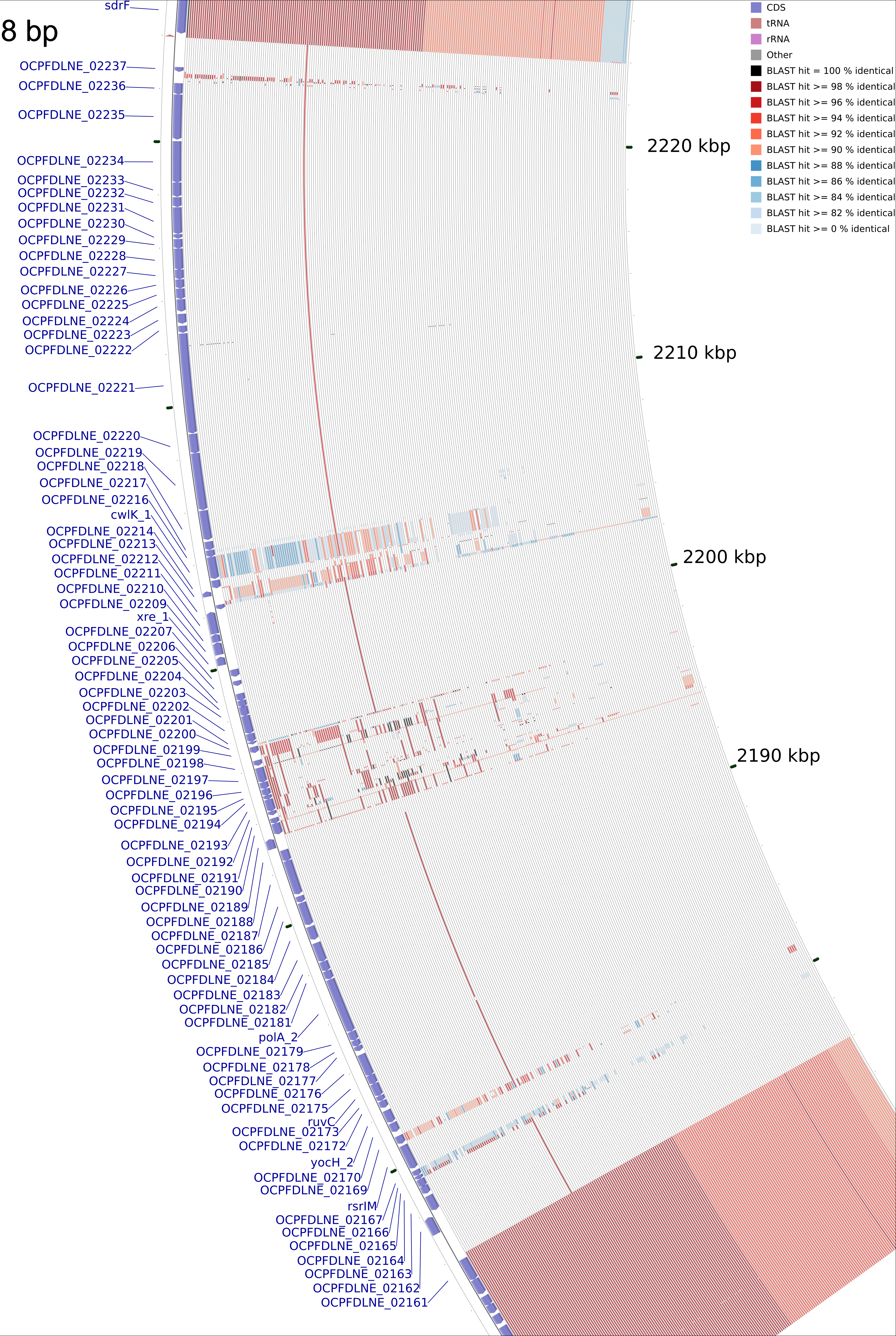

e) Prophage 4: 2559206-2601984 bp

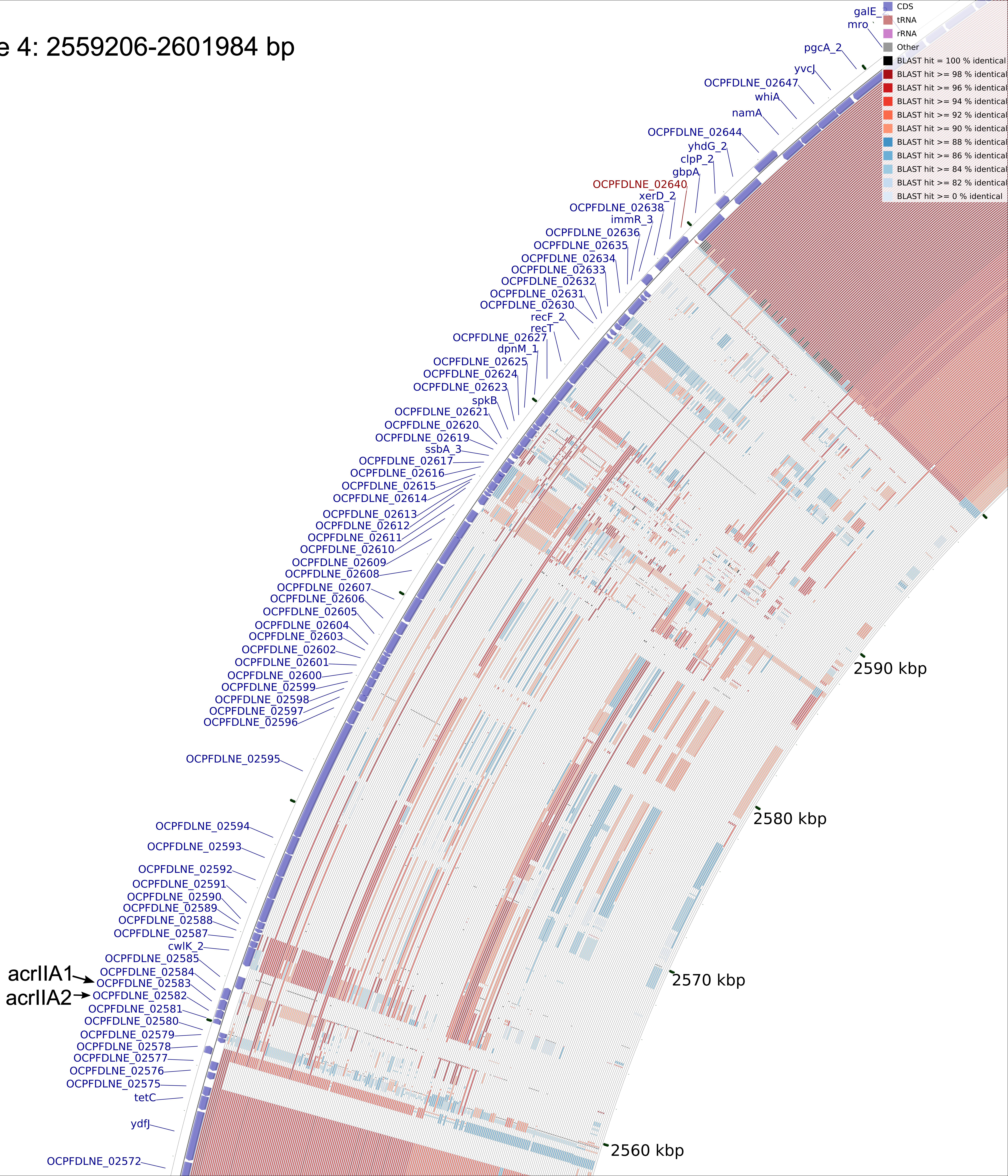

f) Prophage 5: 2729417-2770759 bp

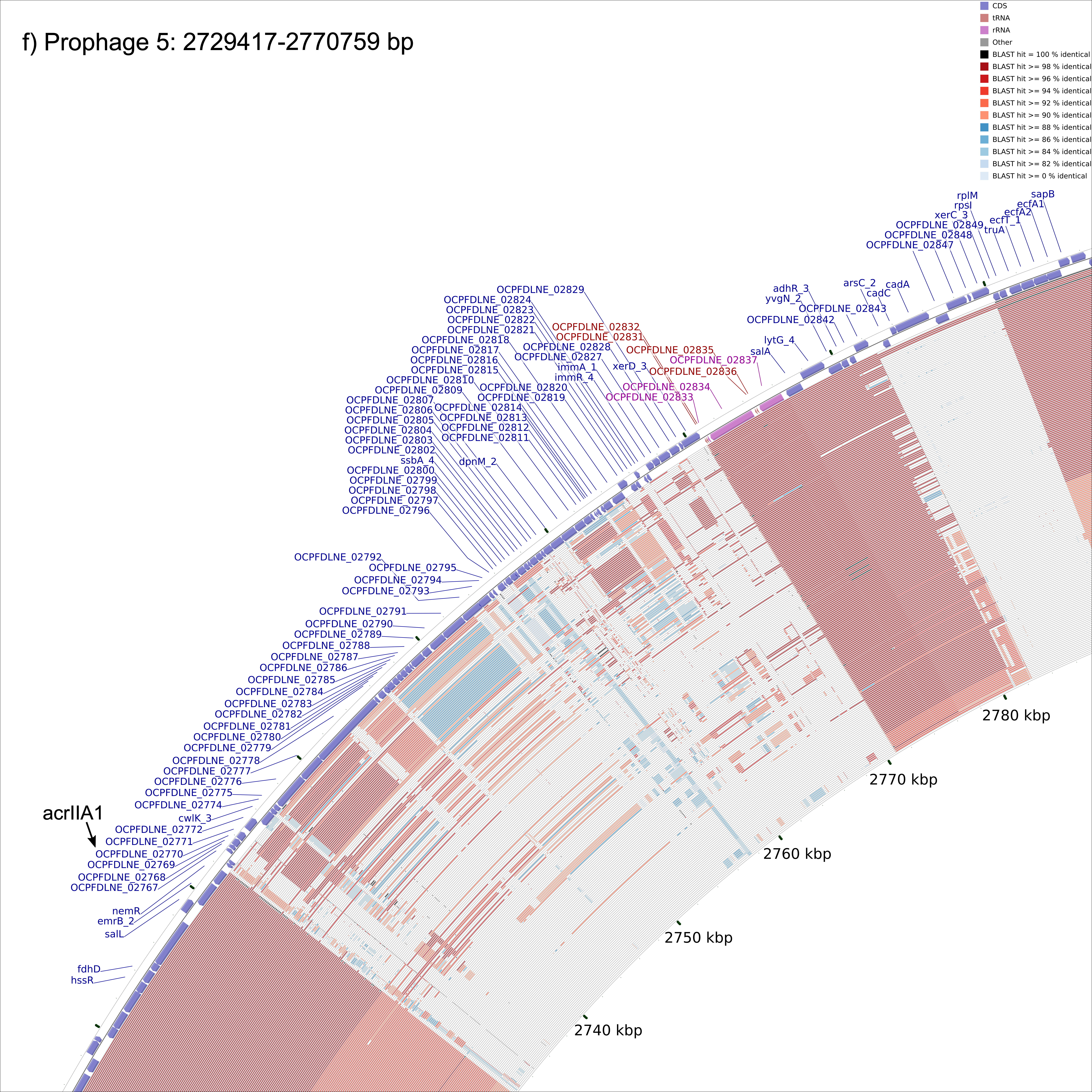

Strains outside to inside

1) NC_021823.1 Listeria monocytogenes strain C1-387
2) NZ_CP021174.1 Listeria monocytogenes strain FORC_057 chromosome
3) NZ_CP045969.1 Listeria monocytogenes strain AUSMDU00007774 chromosome
4) NZ_CP020827.1 Listeria monocytogenes strain CFSAN028538 chromosome
5) NC_017547.1 Listeria monocytogenes Finland 1998
6) NZ_CP007020.1 Listeria monocytogenes serotype 1/2a str. 98-2035
7) NZ_CP007021.1 Listeria monocytogenes serotype 1/2a str. 99-6370
8) 2HF33
9) NZ_CP019167.1 Listeria monocytogenes strain HPB5622
10) NZ_CP006858.1 Listeria monocytogenes serotype 1/2a str. 08-6569
11) NZ_CP006859.1 Listeria monocytogenes serotype 1/2a str. 08-6997
12) NZ_CP006860.1 Listeria monocytogenes serotype 1/2a str. 10-0815
13) NZ_CP007009.1 Listeria monocytogenes serotype 1/2a str. 08-6056
14) NC_013766.2 Listeria monocytogenes 08-5578
15) NZ_CP028183.1 Listeria monocytogenes strain CFSAN054109 chromosome
16) NC_017545.1 Listeria monocytogenes J0161
17) NZ_CP019614.1 Listeria monocytogenes strain 10-092876-1559 LM1
18) NZ_CP021325.1 Listeria monocytogenes strain NH1 chromosome
19) NZ_CP023752.1 Listeria monocytogenes strain AT3E chromosome
20) NZ_CP032669.1 Listeria monocytogenes strain 52869 chromosome
21) NZ_CP025560.1 Listeria monocytogenes strain PIR00545 chromosome
22) NZ_CP013722.1 Listeria monocytogenes strain Lm 3163 chromosome
23) C7
24) NZ_CP019164.1 Listeria monocytogenes strain HPB2088
25) NZ_CP006861.1 Listeria monocytogenes serotype 1/2a str. 10-1047
26) NZ_CP007017.1 Listeria monocytogenes serotype 1/2a str. 10-1046
27) NZ_CP009258.1 Listeria monocytogenes strain Lm60 chromosome
28) NZ_CP018685.1 Listeria monocytogenes strain HPB913 chromosome
29) NZ_CP019170.1 Listeria monocytogenes strain CFSAN042079
30) NZ_CP019165.1 Listeria monocytogenes strain HPB5415
31) NZ_CP007019.1 Listeria monocytogenes serotype 1/2a str. 95-0093
32) NZ_CP007007.1 Listeria monocytogenes serotype 1/2a str. 02-5993
33) NZ_CP007008.1 Listeria monocytogenes serotype 1/2a str. 04-5457
34) NZ_CP007010.1 Listeria monocytogenes serotype 1/2a str. 08-7374
35) NZ_CP007018.1 Listeria monocytogenes serotype 1/2a str. 10-1321
36) NZ_CP008837.1 Listeria monocytogenes serotype 1/2a str. 10-5024
37) NZ_CP008836.1 Listeria monocytogenes serotype 1/2a str. 10-0814
38) NC_013768.1 Listeria monocytogenes 08-5923
39) NZ_HG813249.1 Listeria monocytogenes 6179 chromosome sequence
40) NZ_CP006862.1 Listeria monocytogenes serotype 1/2a str. 88-0478
41) NZ_CP007011.1 Listeria monocytogenes serotype 1/2a str. 08-7669
42) NZ_HG813247.1 Listeria monocytogenes R479a chromosome
43) NZ_CP013724.1 Listeria monocytogenes strain Lm N1546 chromosome
44) EGD-e
45) NZ_CP023861.1 Listeria monocytogenes EGD-e chromosome
46) NZ_CP007170.1 Listeria monocytogenes serotype 1/2a str. 10-0812 strain 1/2a chromosome
47) NZ_CP007171.1 Listeria monocytogenes serotype 1/2a str. 10-0813
48) NZ_CP019618.1 Listeria monocytogenes strain 10-092876-0731 LM5
49) MB5
50) NZ_CP007688.1 Listeria monocytogenes strain L1846
51) NZ_CP023754.1 Listeria monocytogenes strain AL4E chromosome
52) NZ_CP013723.1 Listeria monocytogenes strain Lm 3136 chromosome
53) NZ_LT985476.1 Listeria monocytogenes isolate LMNC088 complete genome chromosome 1
54) NZ_CP025442.1 Listeria monocytogenes strain MF4562 chromosome
55) NZ_CP048400.1 Listeria monocytogenes strain AUF chromosome
56) NZ_CP025443.1 Listeria monocytogenes strain MF4545 chromosome
57) NZ_CP011345.1 Listeria monocytogenes strain FW040025
58) NZ_CP025222.1 Listeria monocytogenes strain ATCC 51775 chromosome
59) NZ_CP007685.1 Listeria monocytogenes strain L2676
60) NZ_CP025568.1 Listeria monocytogenes strain PIR00540 chromosome
61) NC_021837.1 Listeria monocytogenes strain J2-031
62) NZ_CP025440.1 Listeria monocytogenes strain MF6172 chromosome
63) NC_018588.1 Listeria monocytogenes SLCC2372
64) NZ_CP025567.1 Listeria monocytogenes strain ATCC 51779 chromosome
65) NC_018589.1 Listeria monocytogenes SLCC2479
66) NZ_CP019617.1 Listeria monocytogenes strain 10-092876-0055 LM4
67) NZ_CP025438.1 Listeria monocytogenes strain MF4697 chromosome
68) NZ_CP029372.1 Listeria monocytogenes strain 2018TE5305-1-4 chromosome
69) NZ_CP014790.1 Listeria monocytogenes strain 2015TE24968 chromosome
70) NC_017546.1 Listeria monocytogenes FSL R2-561
71) NZ_CP025259.1 Listeria monocytogenes strain MF4624 chromosome
72) NZ_CP007194.1 Listeria monocytogenes serotype 1/2c str. 10-5025
73) NZ_CP007195.1 Listeria monocytogenes serotype 1/2c str. 10-5026
74) NZ_CP020830.1 Listeria monocytogenes strain MOD1_LS152 chromosome
75) NZ_CP041211.1 Listeria monocytogenes strain LMP18-H2446 chromosome
76) NZ_CP025082.1 Listeria monocytogenes strain MF4626 chromosome
77) NZ_CP045970.1 Listeria monocytogenes strain AUSMDU00000235 chromosome
78) NC_017544.1 Listeria monocytogenes 10403S
79) NZ_CP020828.1 Listeria monocytogenes strain CFSAN022990 chromosome
80) NZ_CP007196.1 Listeria monocytogenes serotype 3c str. 10-5027
81) NZ_CP020833.1 Listeria monocytogenes strain CFSAN004330 chromosome
82) NC_018593.1 Listeria monocytogenes SLCC7179
83) NZ_CP025221.1 Listeria monocytogenes strain PIR00543 chromosome
84) NZ_CP019621.1 Listeria monocytogenes strain 10-092876-1235 LM8
85) NZ_CP013919.1 Listeria monocytogenes strain IZSAM_Lm_15_17439_A144
86) NZ_CP019623.1 Listeria monocytogenes strain 10-092876-1763 LM10
87) NZ_CP038642.1 Listeria monocytogenes strain N12-0935 chromosome
88) NZ_CP007197.1 Listeria monocytogenes serotype 1/2a str. 10-4754
89) NZ_CP007198.1 Listeria monocytogenes serotype 1/2a str. 10-4758
90) NZ_CP011397.1 Listeria monocytogenes strain CFSAN007956
91) NZ_CP007160.1 Listeria monocytogenes WSLC1001 chromosome
92) NC_022568.1 Listeria monocytogenes EGD
93) NZ_LR134397.1 Listeria monocytogenes strain NCTC7973 genome assembly
94) NZ_CP007689.1 Listeria monocytogenes strain L2074
95) NC_018592.1 Listeria monocytogenes SLCC5850
96) NZ_CP007687.1 Listeria monocytogenes strain L2625
97) NZ_CP020832.1 Listeria monocytogenes strain PNUSAL000096 chromosome
98) NZ_CP045972.1 Listeria monocytogenes strain AUSMDU00000224 chromosome
99) NZ_CP007684.1 Listeria monocytogenes strain L2626
100) NZ_CP020831.1 Listeria monocytogenes strain PNUSAL000144 chromosome
101) NZ_CP014261.1 Listeria monocytogenes strain 2015TE19005-1355
102) NZ_LT906436.1 Listeria monocytogenes strain NCTC10357 chromosome 1
103) RO4
104) NZ_CP033737.1 Listeria monocytogenes strain FDAARGOS_555 chromosome
105) NZ_CP046362.1 Listeria monocytogenes strain N843_15 chromosome
106) NZ_CP046361.1 Listeria monocytogenes strain N843_10 chromosome
107) AB120
108) NZ_CP007199.1 Listeria monocytogenes serotype 1/2a str. 10-0933
109) NZ_CP007200.1 Listeria monocytogenes serotype 1/2a str. 10-0934
110) AB199
111) NZ_CP029175.1 Listeria monocytogenes strain NCCP 15743 chromosome
112) NZ_CP044429.1 Listeria monocytogenes serotype 4b str. LIS0087 chromosome
113) NC_021826.1 Listeria monocytogenes strain N1-011A
114) NZ_CP014252.1 Listeria monocytogenes strain CFSAN023459
115) NZ_CP007168.1 Listeria monocytogenes serotype 1/2b str. 10-0810
116) NZ_CP007169.1 Listeria monocytogenes serotype 1/2b str. 10-0811 strain 1/2b chromosome
117) NZ_LR698978.1 Listeria monocytogenes isolate MGYG-HGUT-02325 genome assembly
118) NZ_CP016213.1 Listeria monocytogenes strain CFSAN029793
119) NZ_CP023050.1 Listeria monocytogenes strain FDA00011238 chromosome
120) NZ_CP023052.1 Listeria monocytogenes strain FDA00006905 chromosome
121) NZ_CP022020.1 Listeria monocytogenes strain FDA00006907 chromosome
122) NC_021838.1 Listeria monocytogenes strain R2-502
123) NZ_CP041213.1 Listeria monocytogenes strain LMP18-H8393 chromosome
124) NZ_CP032671.1 Listeria monocytogenes strain 52859 chromosome
125) NZ_CP019615.1 Listeria monocytogenes strain 10-092876-0168
126) NZ_CP014250.1 Listeria monocytogenes strain CFSAN010068
127) NZ_CP045749.1 Listeria monocytogenes strain CFSAN008100 chromosome
128) NZ_CP011398.2 Listeria monocytogenes strain CFSAN008100 chromosome
129) NZ_CP019622.1 Listeria monocytogenes strain 10-092876-0145 LM9
130) NZ_CP019624.1 Listeria monocytogenes strain 10-092876-1016 LM11
131) NZ_CP015593.1 Listeria monocytogenes strain ICDC-LM188
132) NZ_CP031141.1 Listeria monocytogenes strain NCCP 14714 chromosome
133) NC_021824.1 Listeria monocytogenes strain J2-064
134) NZ_CP016629.1 Listeria monocytogenes strain FORC_049
135) NC_018587.1 Listeria monocytogenes strain SLCC2755
136) NZ_CP007686.1 Listeria monocytogenes strain L2624
137) NZ_CP020774.1 Listeria monocytogenes strain H34 chromosome
138) NC_018586.1 Listeria monocytogenes SLCC2540
139) NZ_CP020022.1 Listeria monocytogenes strain LI0521 chromosome
140) ScottA
141) NZ_CP025220.1 Listeria monocytogenes strain PIR00546 chromosome
142) NZ_CP025565.1 Listeria monocytogenes strain PIR00544 chromosome
143) NC_021829.4 Listeria monocytogenes J1-220
144) NC_021840.1 Listeria monocytogenes strain J1926
145) NC_021827.1 Listeria monocytogenes strain J1817
146) NC_021839.1 Listeria monocytogenes strain J1776
147) NC_021830.2 Listeria monocytogenes J1816
148) NZ_CP028333.1 Listeria monocytogenes strain CFSAN054108 chromosome
149) NZ_CP041014.1 Listeria monocytogenes strain FDAARGOS_607 chromosome
150) NZ_CP031674.1 Listeria monocytogenes strain CIIMS-NV-3 chromosome
151) NZ_CP045746.1 Listeria monocytogenes strain CFSAN023468 chromosome
152) NZ_CP012021.1 Listeria monocytogenes strain CFSAN023463
153) NZ_CP045745.1 Listeria monocytogenes strain CFSAN023469 chromosome
154) NZ_CP045748.1 Listeria monocytogenes strain CFSAN023464 chromosome
155) NZ_CP045747.1 Listeria monocytogenes strain CFSAN023465 chromosome
156) NZ_CP045751.1 Listeria monocytogenes CFSAN002349 chromosome
157) NZ_CP007459.1 Listeria monocytogenes serotype 4b str. 02-1103
158) NZ_CP007460.1 Listeria monocytogenes serotype 4b str. 02-1289
159) NZ_CP007461.1 Listeria monocytogenes serotype 4b str. 02-1792
160) NZ_CP019625.1 Listeria monocytogenes strain 10-092876-0769 LM12
161) NZ_CP007462.1 Listeria monocytogenes serotype 4b str. 02-6680
162) NZ_CP008821.1 Listeria monocytogenes serotype 4b str. 02-6679
163) NC_021825.1 Listeria monocytogenes strain J1-108
164) NZ_CP007526.1 Listeria monocytogenes serotype 4b str. 81-0592
165) NZ_CP007525.1 Listeria monocytogenes serotype 4b str. 81-0558
166) NZ_CP007167.1 Listeria monocytogenes serotype 4b str. 10-0809
167) NZ_CP006874.1 Listeria monocytogenes serotype 4b str. 81-0861
168) NZ_CP019619.1 Listeria monocytogenes strain 10-092876-1155 LM6
169) NZ_CP007600.1 Listeria monocytogenes strain CFSAN006122
170) NZ_CP026043.1 Listeria monocytogenes strain FDAARGOS_58 chromosome
171) NZ_CP035187.1 Listeria monocytogenes strain N16-0044 chromosome
172) NZ_CP010346.1 Listeria monocytogenes strain IZSAM_Lm_hs2008 chromosome
173) NZ_CP007583.1 Listeria monocytogenes strain XYSN
174) NZ_CP032672.1 Listeria monocytogenes strain 52854 chromosome
175) NC_012488.1 Listeria monocytogenes serotype 4b str. CLIP 80459
176) NZ_LT985475.1 Listeria monocytogenes isolate JF5861 complete genome chromosome 1
177) NZ_CP011004.1 Listeria monocytogenes strain N2306 isolate Human listeriosis chromosome
178) NC_018642.1 Listeria monocytogenes L312
179) NZ_CP040988.1 Listeria monocytogenes strain FDAARGOS_778 chromosome
180) NZ_CP031476.1 Listeria monocytogenes strain M13455 chromosome
181) NZ_CP025219.1 Listeria monocytogenes strain ATCC 13932 chromosome
182) NZ_CP013288.1 Listeria monocytogenes ATCC 19117 strain WSLC 1033 chromosome
183) NZ_CP013289.1 Listeria monocytogenes strain WSLC 1047 chromosome
184) NZ_CP019616.1 Listeria monocytogenes strain 10-092876-1063 LM3
185) NZ_CP013285.1 Listeria monocytogenes strain WSLC 1018 chromosome
186) F2365
187) NC_019556.1 Listeria monocytogenes serotype 4b str. LL195
188) NZ_CP007492.1 Listeria monocytogenes strain NE isolate dc0214 chromosome
189) NC_018585.1 Listeria monocytogenes SLCC2378
190) NZ_CP009897.1 Listeria monocytogenes strain NTSN chromosome
191) NZ_CP007210.1 Listeria monocytogenes WSLC1042 chromosome
192) NZ_CP023321.1 Listeria monocytogenes strain CIIMS-PH-1 chromosome
193) NC_017728.1 Listeria monocytogenes 07PF0776
194) NZ_LT985474.1 Listeria monocytogenes isolate JF5203_1 chromosome 1
195) NZ_CP033612.1 Listeria monocytogenes strain LMJF5203 chromosome
196) NZ_CP019620.1 Listeria monocytogenes strain 10-092876-1547 LM7
197) NZ_CP033738.1 Listeria monocytogenes strain FDAARGOS_554 chromosome
198) NZ_CP013287.1 Listeria monocytogenes strain WSLC 1020 chromosome
199) NZ_CP013286.1 Listeria monocytogenes strain WSLC 1019 chromosome
200) NC_018590.1 Listeria monocytogenes SLCC2376
201) NZ_CP032668.1 Listeria monocytogenes strain 52873 chromosome
202) NC_017529.1 Listeria monocytogenes L99
203) NZ_CP048401.1 Listeria monocytogenes strain 4/52-1953 chromosome
204) NC_017537.1 Listeria monocytogenes M7
205) NC_011660.1 Listeria monocytogenes HCC23
206) NZ_CP009242.1 Listeria monocytogenes strain LM850658
207) NZ_CP032670.1 Listeria monocytogenes strain 52860 chromosome
208) NZ_CP032673.1 Listeria monocytogenes strain 52330 chromosome
209) NZ_LR134398.1 Listeria monocytogenes strain NCTC7974 chromosome 1
